# Wafer-Scaled III-Nitrides Nanowire Photocathodes Enabled by Synergistic Dual-Electron Extraction for Efficient Solar-to-Hydrogen Conversion

**DOI:** 10.1007/s40820-026-02186-9

**Published:** 2026-04-17

**Authors:** Xudong Yang, Yuying Liu, Wei Chen, Tianle Zhang, Wengang Gu, Xin Liu, Yuanmin Luo, Zhixiang Gao, Yang Li, Menglong Wang, Weiyi Wang, Ran Long, Wei Hu, Jiajie Xu, Haiding Sun

**Affiliations:** 1 iGaN Laboratory, School of Microelectronics, University of Science and Technology of China, Hefei, 230026 Anhui People’s Republic of China; 2 National Synchrotron Radiation Laboratory, University of Science and Technology of China, Hefei, 230027 People’s Republic of China; 3 Department of Chemical Physics, Hefei National Laboratory for Physical Science at the Microscale, University of Science and Technology of China, Hefei, 230027 People’s Republic of China; 4 Microbial Development and Metabolic Engineering Laboratory, School of Marine Science, Ningbo University, Ningbo, 315211 People’s Republic of China

**Keywords:** Photoelectrochemistry, Wafer-scale water splitting, InGaN nanowires, Internal–external synergy, Dual-electron extraction

## Abstract

Efficient, durable, and scalable photocathodes are indispensable for large-scale solar-to-hydrogen production. Notably, single-junction semiconductor photocathodes are attractive due to their structural simplicity, cost-effectiveness, and mature fabrication, yet they usually exhibit intrinsically poor carrier extraction efficiency. To address this challenge, we propose a synergistic “dual-electron extraction” strategy that fully unleashes the hydrogen evolution potential of single-junction p-InGaN nanowires. Remarkably, the optimized p-InGaN photocathode achieves a photocurrent density of −3.40 mA cm^−2^ at 0 V vs. RHE—representing a 37.8-fold enhancement over the pristine device—with an onset potential of 0.82 V vs. RHE, while sustaining stable hydrogen generation for over 300 h without additional protective layers. Specifically, an electron-blocking layer was incorporated within p‑InGaN nanowires to suppress electron backflow toward the substrate and promote transport to the nanowire/electrolyte interface. Furthermore, surface anion doping in InGaN nanowires significantly enhances the band bending of InGaN, which promotes interfacial electron transfer while simultaneously optimizing hydrogen adsorption energy, thereby accelerating the hydrogen evolution reaction rate. The proposed synergistic dual-electron extraction strategy markedly improves the electron utilization efficiency of single-junction InGaN nanowires, providing a novel pathway to address the intrinsic limitations of wafer-scale III-V nitride photoelectrodes.

**Supplementary Information:**

The online version contains supplementary material available at 10.1007/s40820-026-02186-9.

## Highlights

- A scalable internal–external synergistic dual-electron extraction strategy is proposed, enabling rapid bulk-surface-electrolyte electron transport in single-junction p-InGaN nanowire for highly efficient photoelectrochemical hydrogen evolution.
- Optimized single-junction p-InGaN photoelectrode exhibits a superior onset potential of 0.82 V vs. RHE and maintains continuous hydrogen generation for more than 300 h without protective coatings.
- This material platform and optimization strategy possess wafer-scale scalability and manufacturing compatibility, providing new technical insights for photoelectrochemical hydrogen production with industrial application value.

**Supplementary Information:**

The online version contains supplementary material available at 10.1007/s40820-026-02186-9.

## Graphical abstract

**Figure:**
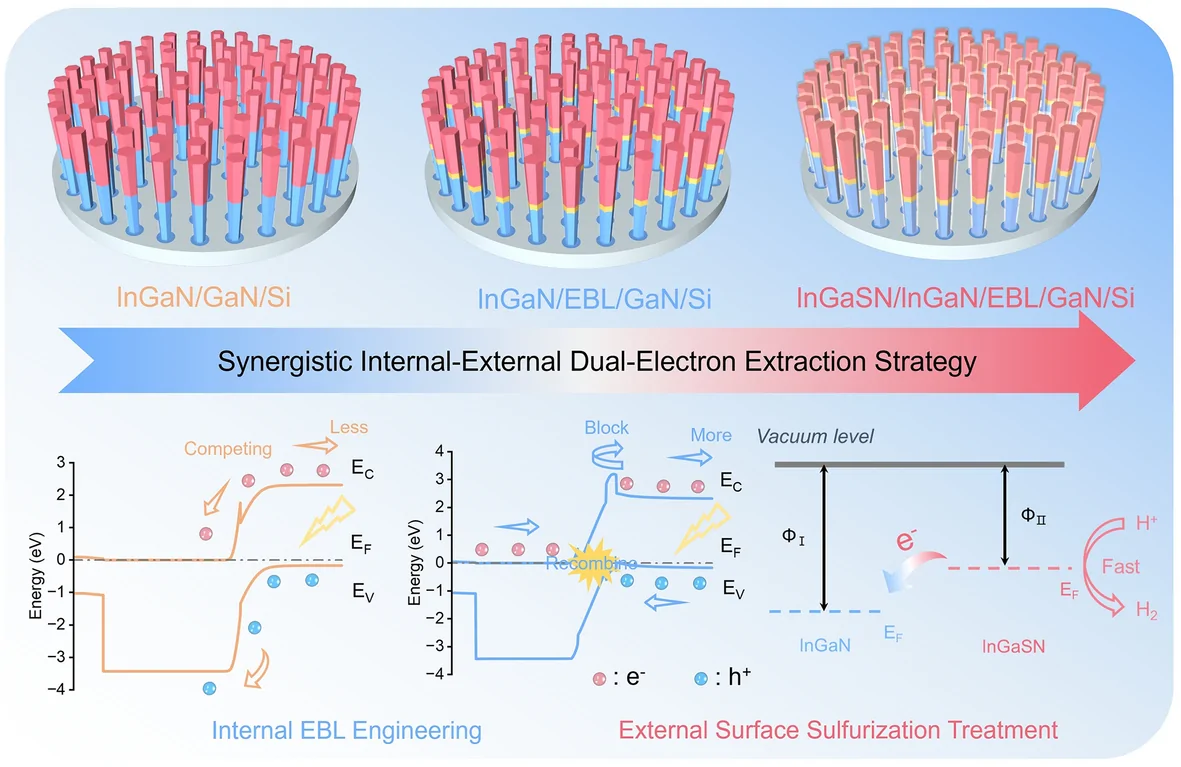

## Introduction

Solar-driven water splitting for hydrogen production is widely recognized as a key pathway toward carbon neutrality, owing to its sustainability and environmental advantages [1, 2]. Among the various strategies, photoelectrochemical (PEC) water splitting has garnered significant attention due to its straightforward device architecture and direct energy conversion process [3–5]. In PEC systems, semiconductor-based photoelectrodes play a pivotal role in determining both the hydrogen evolution rate and the overall device stability. Notably, among the numerous semiconductor candidates (e.g., Si, Cu_2_O, and III–V compounds), In_x_Ga_(1−x)_N nanowires (NWs) have emerged as promising candidates because of their unique material properties and advantages associated with their one-dimensional structure [6–9]. Specifically, In_x_Ga_(1−x)_N NWs demonstrate outstanding chemical stability under acidic conditions, highly efficient photon absorption, and an suitable bandgap for water splitting [9–11]. Moreover, their well-established fabrication techniques and scalability for wafer-level production [6, 12] ensure high reproducibility, cost-effectiveness, and suitability for large-scale manufacturing, thereby promising to meet the demands of future industrial deployment. Nevertheless, conventional In_x_Ga_(1−x)_N NW-based photoelectrodes continue to face critical challenges, including limited carrier separation efficiency and insufficient surface reaction activity, which impede the realization of efficient PEC hydrogen production and restrict their broader deployment.

In recent years, extensive efforts have been directed toward optimizing the performance of In_x_Ga_(1−x)_N NW-based photoelectrodes. On one front, the construction of homojunctions or heterojunctions to generate built-in electric fields [13–15], together with the design of graded energy-band structures [16], has been employed to enhance the separation and transport of photogenerated electron–hole pairs within the NWs. On another front, surface passivation [17] and cocatalyst loading [18–21] have been utilized to accelerate charge transfer across the NW/electrolyte interface and to enhance surface reaction activity. Overall, the hydrogen evolution performance of In_x_Ga_(1−x)_N NW photocathodes is fundamentally governed by internal and interfacial charge transport, together with surface catalytic activity. High-performance In_x_Ga_(1−x)_N NW photocathodes must satisfy three essential requirements: (i) efficient separation and transport of photogenerated carriers within the NWs, (ii) rapid electron transfer across the NW/electrolyte interface, and (iii) strong catalytic activity for hydrogen evolution at the NW surface [5, 22, 23]. Therefore, the development of a synergistic strategy that concurrently enhances internal and external carrier transport and interfacial reaction activity is critical to unlocking the full potential of In_x_Ga_(1−x)_N nanowire photoelectrodes.

Here, based on wafer-scale single-junction p-InGaN nanowires, which are cost-effective and technologically mature, we report a scalable synergistic dual-electron extraction strategy that establishes a rapid pathway for photogenerated electron transfer from the nanowire bulk to the electrolyte. The optimized p-InGaN nanowire photoelectrodes exhibit efficient and stable hydrogen evolution in acidic environments, delivering a photocurrent density of −3.40 mA cm^−2^ at 0 V vs. RHE—a 37.8-fold enhancement over the pristine electrode—with an onset potential of 0.82 V vs. RHE. Remarkably, the device sustained efficient hydrogen evolution for more than 300 h without employing a surface protection layer. Specifically, an electron-blocking layer (EBL) composed of n^++^-GaN, InGaN, and p^++^-GaN is integrated within the single-junction nanowire. This EBL effectively suppresses the flow of photoelectrons in p-InGaN into the internal circuit while allowing the photoholes in p-InGaN to pass through the layer and reach the circuit. Externally, sulfur-ion doping on the surface of InGaN NWs effectively improves the band bending of InGaN, thereby providing a stronger driving force for electron transfer to the electrolyte. Meanwhile, the doped surface exhibits optimized hydrogen adsorption energy, which accelerates the hydrogen evolution reaction rate. The successful implementation of this internal–external synergistic dual-electron extraction strategy provides new technical insights and experiential support for the future development of large-scale, scalable, and high-performance photocathodes.

## Experimental Section

### Growth of the lnGaN Nanowires

All single-junction one-dimensional nanowires used in the experiment were epitaxially grown on n-Si substrates using plasma-assisted molecular beam epitaxy. The nanowires were synthesized under nitrogen-rich conditions, with a plasma power of 300 W and a nitrogen flow rate of 1.5 sccm. Silicon and magnesium served as dopants for n-type and p-type nanowires, respectively. The n-GaN nanowire segment was grown at a substrate temperature of 730 °C, with a Ga BEP of approximately 7.7 × 10^–8^ Torr. The p-InGaN nanowire segment was grown at a substrate temperature of about 600 °C, with a Ga BEP of 2.4 × 10^–8^ Torr and an In BEP of roughly 2.2 × 10^–8^ Torr. The electron-blocking layer was grown at a substrate temperature of 610 °C and consists of n^++^-GaN, InGaN, and p^++^-GaN.

### Sulfurization Process

The single-junction one-dimensional nanowires were subjected to surface sulfuration via a simple sulfuration process. Approximately 100 mg of high-purity sulfur powder and the samples were placed in the upstream and downstream regions of a quartz tube furnace, respectively. During the sulfuration process, a flowing atmosphere of H_2_-Ar mixed gas (containing 10% high-purity hydrogen) was maintained at a flow rate of 50 sccm. The downstream heating zone was set to 500 °C, while the upstream zone was maintained at 180 °C, with the heating duration fixed at 30 min.

### Photoelectrochemical Measurements

The prepared photocathode was characterized by PEC measurements using a CHI760E electrochemical workstation with a three-electrode setup in a 0.5 M H_2_SO_4_ aqueous electrolyte, under an illumination intensity of AM 1.5G one sun. In this configuration, the photocathode serves as the working electrode, while the Ag/AgCl electrode and platinum mesh are used as the reference and counter electrodes, respectively. The potential of the photocathode relative to the Ag/AgCl electrode (*V*_Ag/AgCl_) was converted to the standard hydrogen electrode (RHE) scale (E) using the Nernst equation, as described below:

1 $$E = V_{{\mathrm{Ag/AgCl}}} + 0.059pH + V_{{\mathrm{Ag/AgCl}}}^{{0}}$$

Here, *E* represents the applied potential on the reversible hydrogen electrode (RHE) scale (*E*_*RHE*_). The standard potential of the Ag/AgCl electrode at room temperature (25 °C) is ${V}_{\mathrm{Ag}/\mathrm{AgCl}}^{0}$ = 0.197 V. Linear sweep voltammetry (LSV) measurements were conducted at an illumination intensity of 100 mW cm^−2^ via a simulated 300 W xenon lamp (PLS-CS300, Beijing Perfectlight) equipped with an AM 1.5 filter. The sample was exposed to light through a quartz window in the cell, and the light intensity was calibrated with a PL-MW2000 (Beijing Perfectlight) Photoradiometer. The potential range for both photoelectrochemical impedance spectroscopy (PEIS) and electrochemical impedance spectroscopy (EIS) measurements was 0 V vs. RHE, with a frequency range of 0.1 to 100,000 Hz. The applied bias photon-to-current efficiency (ABPE) of the photocathode was calculated via the following equation:

2 $$ABPE \left( \% \right) = \frac{{J\left( {V_{RHE} - E_{rev} } \right)}}{{P_{in} }} \times 100\%$$

where *J* refers to the photocurrent density, *E*_rev_ = 0 V vs. RHE, *V*_RHE_ refers to the applied voltage vs. RHE, and *P*_in_ = 100 mW cm^−2^ refers to the incident light intensity. The incident photon-to-current efficiency (IPCE) is calculated via the following equation:

3 $$IPCE \left( \% \right) = \frac{{\left( {1240 \times J} \right)}}{{\lambda \times P_{{{\mathrm{in}}}} }} \times 100\%$$

where *J* represents the photocurrent density (mA cm^−2^), *λ* represents the wavelength of incident light (nm), and *P*_in_ represents the power density (mW cm^−2^) of the incident illumination. The electrochemical double-layer capacitance (*C*_dl_) was calculated by cyclic voltammetry (CV) curves in the region of between 0.587 and 0.687 V vs. RHE with different scanning rates of 40, 60, 80, 100, and 120 mV s^−1^ in 0.5 M H_2_SO_4_ (pH = 0). The evaluate the electrochemical surface area (ECSA) was proportional to the *C*_dl_.

The charge separation efficiency (η_sep_) can be calculated using the following equation:

4 $$\eta_{{{\mathrm{sep}}}} = \frac{{J_{{{\mathrm{Na}}_{{2}} {\mathrm{S}}_{{2}} {\mathrm{O}}_{{8}} }} }}{{J_{{{\mathrm{abs}}}} }} \times 100\%$$

The electron scavenger used was 0.5 M Na_2_S_2_O_8_. The photocurrent density (*J*_abs_) was calculated by integrating the AM 1.5G simulated solar spectrum after determining the band gap using photoluminescence spectrum measurements.

### Material Characterization

Scanning electron microscopy (SEM) was performed using a Hitachi SU8220 system. The morphology of the synthesized nanowires was analyzed via high-resolution transmission electron microscopy (HRTEM) via a Talos F200X system operating at 200 kV. Energy-dispersive X-ray spectroscopy (EDS) was also carried out via the Talos F200X. Photoluminescence (PL) measurements were performed at room temperature using a 266 nm excitation pulse laser, with the PL signal collected by an ultraviolet objective and analyzed using an OceanOptics QE Pro spectrometer. Time-resolved photoluminescence (TRPL) spectra were measured by the TPL-300 (Time-Tech Spectra) with a 230 nm pulsed laser. Synchrotron X-ray absorption spectroscopy (XAS) was acquired on the Photoemission End station at the MCD-A and MCD-B beamline in the National Synchrotron Radiation Laboratory (NSRL) in Hefei, China. X-ray photoelectron spectroscopy (XPS) measurements were conducted using a Thermo Scientific K-Alpha XPS instrument equipped with an Al Kα source (hν = 1486.6 eV). Spectral positions were corrected using adventitious carbon, by shifting the C 1s core-level position to 284.8 eV.

### Density Functional Theory Calculation Details

Total energy calculations were performed within the framework of density functional theory (DFT). The spin-polarized calculations were performed using a projector augmented wave (PAW) 30 method and the Perdew–Burke–Ernzerhof (PBE) [24] exchange–correlation functional within the generalized gradient approximation (GGA) [25], as implemented in the Vienna ab initio simulation package (VASP) [26].

The initial structure of InGaN is derived from the GaN lattice, with an In/Ga ratio of 1:3. Indium atoms are incorporated by uniformly substituting Ga atoms, resulting in a homogeneous alloy structure. The obtained InGaN unit cell was then expanded into a 4 × 2 × 1 supercell to minimize periodic interaction and better represent the bulk-like environment. In addition, the vacuum regions are kept at least 15 Å apart along the c-axis to eliminate the effect of interlayer interactions. Based on this model, several representative sulfur surface doping models were considered, and their details will be elaborated in the subsequent sections. In our calculations, the cutoff energy was set at 400 eV. The shape and volume of the unit cell as well as the atomic positions in the unit cell of each configuration were fully optimized. Atomic positions were relaxed until the forces on each atom reducing to less than 0.01 eV Å^−1^. A Gamma-centered 1 × 1 × 1 k-point mesh was employed for the 4 × 2 × 1 InGaN supercell. The convergence of the k-point sampling and the size of the supercell was rigorously tested.

The relative surface formation energies with respect to the pristine InGaN (10 $\overline{1 }$ 0) surface were evaluated using the chemical potential formalism with necessary modifications for S substitution and computed as:

5 $$E_{{{\mathrm{form}}}} = \frac{1}{A}\left( {E_{{{\mathrm{tot}}}} - E_{{{\mathrm{ref}}}} - \sum\nolimits_{{\mathrm{i}}} {\mu_{{\mathrm{i}}} \Delta n_{{\mathrm{i}}} } } \right)$$

where A is the surface area, E_tot_ is the total energy of the S-substituted configuration, and E_ref_ is the total energy of the pristine N-terminated InGaN slab. Here, ${\mu }_{i}$ is the chemical potential of the *i*th species, and $\Delta {n}_{\mathrm{In}},\Delta {n}_{\mathrm{Ga}},\Delta {n}_{\mathrm{N}}$, and $\Delta {n}_{\mathrm{S}}$ represent the excess or deficit of In, Ga, N, and S atoms with respect to the reference, respectively. Specifically, the In, Ga, and N chemical potentials were assumed to satisfy the equilibrium condition of bulk InGaN, i.e.,

6 $$\mu_{{{\mathrm{In}}}} + \mu_{{{\mathrm{Ga}}}} + \mu_{{\mathrm{N}}} = E_{{{\mathrm{InGaN}}}}$$

where $E_{lnGaN}$ denotes the total energy of bulk InGaN at an In/Ga ratio of 1:3. For sulfur, the chemical potential was referenced to the bulk sulfide phase, such as Ga_2_S_3_, by requiring that

7 $$2\mu_{{{\mathrm{Ga}}}} + 3\mu_{{\mathrm{S}}} = E_{{{\mathrm{Ga}}_{{2}} {\mathrm{S}}_{{3}} }}$$

where ${E}_{{\mathrm{Ga}}_{2}{\mathrm{S}}_{3}}$ is the total energy of monoclinic Ga_2_S_3._ In this framework, the surface formation energy becomes a function of ${\mu }_{\mathrm{Ga}}$ within the stability range of InGaN, while ${\mu }_{\mathrm{S}}$ is constrained by the sulfide reference. This approach allows a direct comparison of the relative stabilities of pristine and different S-substituted surfaces, providing insight into the thermodynamic feasibility of sulfur incorporation at the InGaN surface.

The local density of states (LDOS) extends the concept of the density of states (DOS) by incorporating the spatial distribution of the partial charge density:

8 $$LDOS(E,r) = \frac{{N_{{\mathrm{e}}} \Omega_{{{\mathrm{cell}}}} }}{{\left( {2\pi } \right)^{3} }}\sum\nolimits_{{\mathrm{n}}} {\int_{{{\mathrm{BZ}}}} {\delta \left( {E - \varepsilon_{{\mathrm{n,k}}} } \right)} } P_{{\mathrm{n,k}}} \left( r \right)d^{3} k$$

with ${\mathrm{N}}_{\mathrm{e}}$ is the spin-degeneracy factor, ${\Omega }_{cell}$ is the unit cell volume ( $\Omega_{cell}=L_1 L_2 L_3 \vert\widehat{L}_{3}\cdot(\widehat{L}_{1}\times\widehat{L}_{2})\vert$ and ${L}_{i}$ and ${\hat{L}}_{i}$ are the magnitude and unit vector of the lattice vectors of the unit cell, respectively). ${P}_{n,k}(r)$ stands for the partial charge density which describes the probability ${\left|{\phi }_{n,k}(r)\right|}^{2}$ finding an electron wave function with a given wavevector k and band index n in a given spatial region. The spatially resolved DOS (plane-averaged LDOS) is obtained by averaging the 3D LDOS over the plane spanned by two lattice vectors. This calculation was implemented using the DensityTool package [27], which allows for an efficient integration of VASP-generated electronic states, yielding a distribution along the third vector as a function of energy.

The Gibbs free energy change ($\Delta {\mathrm{G}}_{H}$) for the hydrogen evolution reaction was calculated using the computational hydrogen electrode (CHE) model proposed by Nørskov et al. [28]. Within this framework, the adsorption free energy of H on the surface is expressed as:

9 $$\Delta G_{H} = \Delta E_{H} + \Delta E_{ZPE} - T\Delta S_{H}$$

where $\Delta E_{{\mathrm{H}}} = E_{{\text{slab + H}}} - E_{{\text{slab }}} - \frac{1}{2}E_{{{\mathrm{H}}_{2} }}$ is the adsorption energy of hydrogen relative to molecular hydrogen, $\Delta {E}_{\mathrm{ZPE}}$ is the zero-point energy correction, and $\Delta {S}_{H}$ is the entropy contribution of adsorbed hydrogen relative to ${\mathrm{H}}_{2}( \mathrm{g})$ at standard conditions. *TS* values H_2_ are from previous report [29].

## Results and Discussion

### Internal Optimization—Constructing an Electron-Blocking Layer

**Fig. 1:**
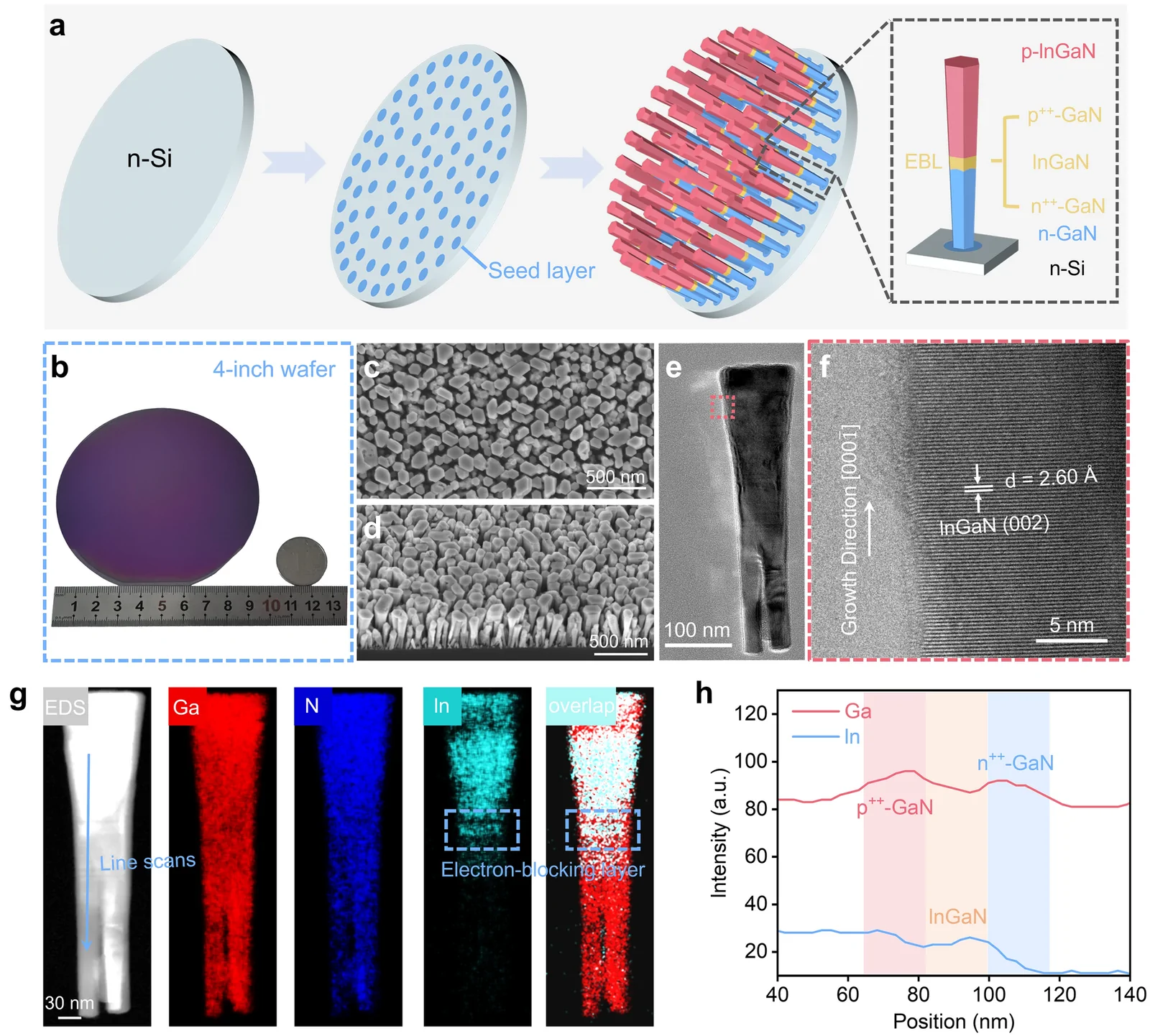
Morphological and structural characterization of wafer-scale p-InGaN/EBL/n-GaN nanowires. **a** Schematic illustration of the fabrication process for the integrated nanowire array. **b** Photograph of the as-fabricated 4-inch wafer. **c** Top-view and **d** 45° tilted-view SEM images of the nanowire array. **e** TEM image of the p-InGaN/EBL/n-GaN nanowire. **f** HRTEM image of the region marked in **e**. **g** EDS elemental mapping of the p-InGaN/EBL/n-GaN nanowire. **h** Corresponding line-scan profiles of Ga and In signals along the direction indicated in (**g**)

The fabrication process of the p-InGaN/EBL/n-GaN NW array, designed as an integrated configuration for photoelectrochemical water splitting, is illustrated in Fig. 1a. Single-junction p-InGaN NWs were first grown on n-Si (111) substrates under nitrogen-rich conditions via a bottom-up plasma-assisted molecular beam epitaxy (MBE) approach. To mitigate defects and dislocations associated with the direct growth of p-InGaN NWs on n-Si and to prevent phase separation, the NWs were integrated with the silicon substrate via a bottom n-GaN segment [6, 30]. Leveraging the precise interface control afforded by MBE, an EBL composed of n^++^-GaN, InGaN, and p^++^-GaN was incorporated into the single-junction p-InGaN NWs to enhance electron–hole separation and transport. Figure 1b illustrates a 4-inch wafer (≈ 10 cm in diameter), demonstrating the scalability of InGaN NW wafer production. Detailed growth procedures are provided in the Methods section. Scanning electron microscopy (SEM) images (Fig. 1c, d) reveal that the NWs are uniformly aligned vertically on the Si substrate. Transmission electron microscopy (TEM) analysis (Fig. 1e) revealed that the NW has a length of ~ 430 nm and a diameter of ~ 80 nm, dimensions smaller than the carrier diffusion length of p-InGaN, thereby effectively suppressing intrinsic carrier recombination [31, 32]. High-resolution TEM (HRTEM) (Fig. 1f) confirmed that the NW grew along the [000 $\overline{1 }$] direction, with lattice fringes corresponding to the (002) plane of InGaN and a spacing of d = 2.60 Å, indicative of high crystallinity. Further structural characterization was performed using dark-field TEM and energy-dispersive spectroscopy (EDS) elemental mapping (Fig. 1g), which clearly distinguished the InGaN segment in the upper NW region, the GaN segment at the base, and the InGaN layer within the EBL. A line scan performed along the direction indicated in Fig. 1g reveals distinct variations in the Ga and In signals across different regions of the n^++^-GaN/InGaN/p^++^-GaN EBL (Fig. 1h), further confirming the presence of the EBL. Collectively, these results demonstrate the successful epitaxial growth of high-quality p-InGaN NWs on silicon wafers incorporating the designed electron-blocking layer structure.

### Performance and Regulatory Mechanism after the Internal Construction of the EBL

**Fig. 2:**
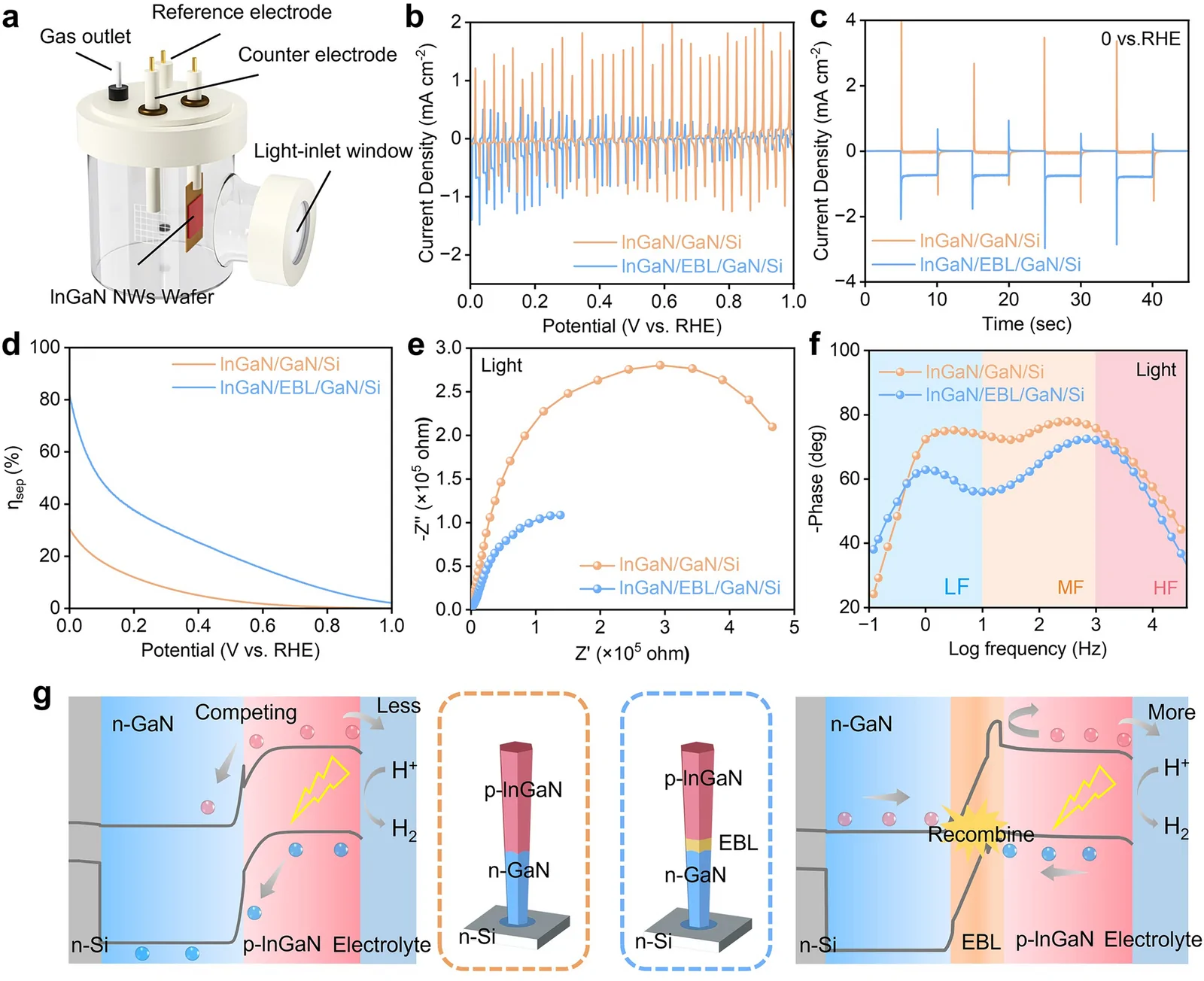
Characterization of performance and mechanism analysis regarding internal optimization. **a** Schematic illustration of the three-electrode PEC measurement setup. **b** Chopped J-V curves of InGaN/GaN/Si and InGaN/EBL/GaN/Si. **c** I-t curves of InGaN/GaN/Si and InGaN/EBL/GaN/Si at 0 V vs. RHE. **d** η_sep_ of InGaN/GaN/Si and InGaN/EBL/GaN/Si. **e** Nyquist plots and **f** corresponding Bode plots of InGaN/GaN/Si and InGaN/EBL/GaN/Si measured under irradiation. **g** Schematic illustration of the photogenerated carrier transport behavior of the InGaN/GaN/Si and InGaN/EBL/GaN/Si photocathodes under illumination (red spheres denote electrons, while blue spheres indicate holes)

To investigate the role of the EBL in the PEC water splitting process of p-InGaN/EBL/n-GaN nanowires, we conducted a comprehensive performance evaluation of InGaN/GaN/Si and InGaN/EBL/GaN/Si using a three-electrode PEC setup (Fig. 2a). The chopped photocurrent–voltage (J-V) curves under simulated sunlight (AM 1.5G, 100 mW cm^−2^) in 0.5 M H_2_SO_4_ are shown in Fig. 2b. Compared with InGaN/GaN/Si, the PEC hydrogen evolution performance of InGaN/EBL/GaN/Si was significantly enhanced. At 0 V vs. RHE, the photocurrent density reached −0.79 mA cm^−2^, corresponding to an 8.8-fold improvement over InGaN/GaN/Si (−0.09 mA cm^−2^) (Fig. 2c). To further elucidate the role of the EBL, Na_2_S_2_O_8_ was employed as an electron sacrificial agent. As a strong electron acceptor with rapid reduction kinetics, Na_2_S_2_O_8_ ensures that photogenerated electrons reaching the semiconductor surface are fully utilized. Thus, the separation and transport efficiency of internal charge carriers can be quantified by the ratio of the measured photocurrent (J_sep_) in 0.5 M H_2_SO_4_ + 0.5 M Na_2_S_2_O_8_ to the ideal photocurrent of the photocathode (J_abs_), where η_sep_ = J_sep_/J_abs_ [33]. The ideal photocurrent of the photocathode was calculated to be -6.299 mA cm^−2^ using the AM 1.5G simulated solar spectrum (Fig. S1). As shown in Figs. 2d and S2, InGaN/EBL/GaN/Si has a substantially higher η_sep_ across a range of potentials compared with InGaN/GaN/Si. At 0 V vs. RHE, the η_sep_ of InGaN/EBL/GaN/Si is approximately 2.7 times greater, providing strong evidence that the EBL plays a critical role in enhancing the separation and transport of internal charge carriers.

To further elucidate the mechanism of internal charge carrier separation and transport upon the introduction of the EBL, we present the photoelectrochemical impedance spectroscopy (PEIS) results of InGaN/GaN/Si and InGaN/EBL/GaN/Si and simulated the corresponding band diagrams using Advanced Physical Models of Semiconductor Devices (APSYS) provided by Crosslight (Fig. S3). Figure 2e shows the Nyquist plots of both structures under irradiation. In general, a larger semicircle diameter reflects a higher charge transfer resistance (R_ct_). Accordingly, the results suggest that inserting the EBL significantly lowers R_ct_ [8, 34]. For deeper analysis, the Bode plots of the two structures under irradiation are shown in Fig. 2f. In Bode plots, the magnitude of the phase angle reflects the extent of charge participation in the transfer process, with smaller phase angles indicating a greater number of charges involved [33]. In semiconductor/electrolyte systems under illumination, the low-frequency (LF) region of PEIS primarily reflects charge transfer during the electrochemical reaction at the interface, while the mid-frequency (MF) region mainly corresponds to carrier transport and recombination within the semiconductor [35, 36]. As shown in Fig. 2f, the phase angles of InGaN/EBL/GaN/Si are consistently smaller than those of InGaN/GaN/Si at both LF and MF regions, demonstrating that the introduction of the EBL enhances the separation and transport of photogenerated electrons and holes within the photocathode. Furthermore, the Bode plots of InGaN/GaN/Si and InGaN/EBL/GaN/Si under dark conditions (Fig. S4) exhibit minimal differences, suggesting comparable surface activities. Therefore, the observed variations in the PEIS are attributed primarily to the influence of the internal structure. The role of the EBL can be further clarified by the band diagram in Fig. 2g. In single-junction p-InGaN photocathodes, p-InGaN functions both as a light absorber and as an active site for the hydrogen evolution reaction. Consequently, photogenerated electrons in p-InGaN can directly migrate to the p-InGaN/electrolyte interface to participate in hydrogen evolution. However, the presence of n-GaN at the NW base generates an internal electric field between n-GaN and p-InGaN, which diverts part of the photogenerated electrons into the internal circuit rather than toward the electrolyte interface, thereby reducing the electron population available for hydrogen evolution. Moreover, the photogenerated holes in p-InGaN must overcome the high potential barrier imposed by the n-GaN base to flow into the internal circuit. This sluggish hole transport increases the likelihood of recombination with photogenerated electrons, further diminishing the electron density at the p-InGaN/electrolyte interface. By introducing an EBL into the single-junction p-InGaN nanowire, photogenerated electrons are more effectively directed to the p-InGaN/electrolyte interface. Simultaneously, the EBL facilitates rapid hole transport into the internal circuit, thereby enhancing electron–hole separation efficiency. In summary, inserting an EBL between p-InGaN and n-GaN not only increases the number of photogenerated electrons reaching the electrolyte interface but also improves the hole transport efficiency within the internal circuit.

### External Optimization—Constructing the InGaSN Surface

**Fig. 3:**
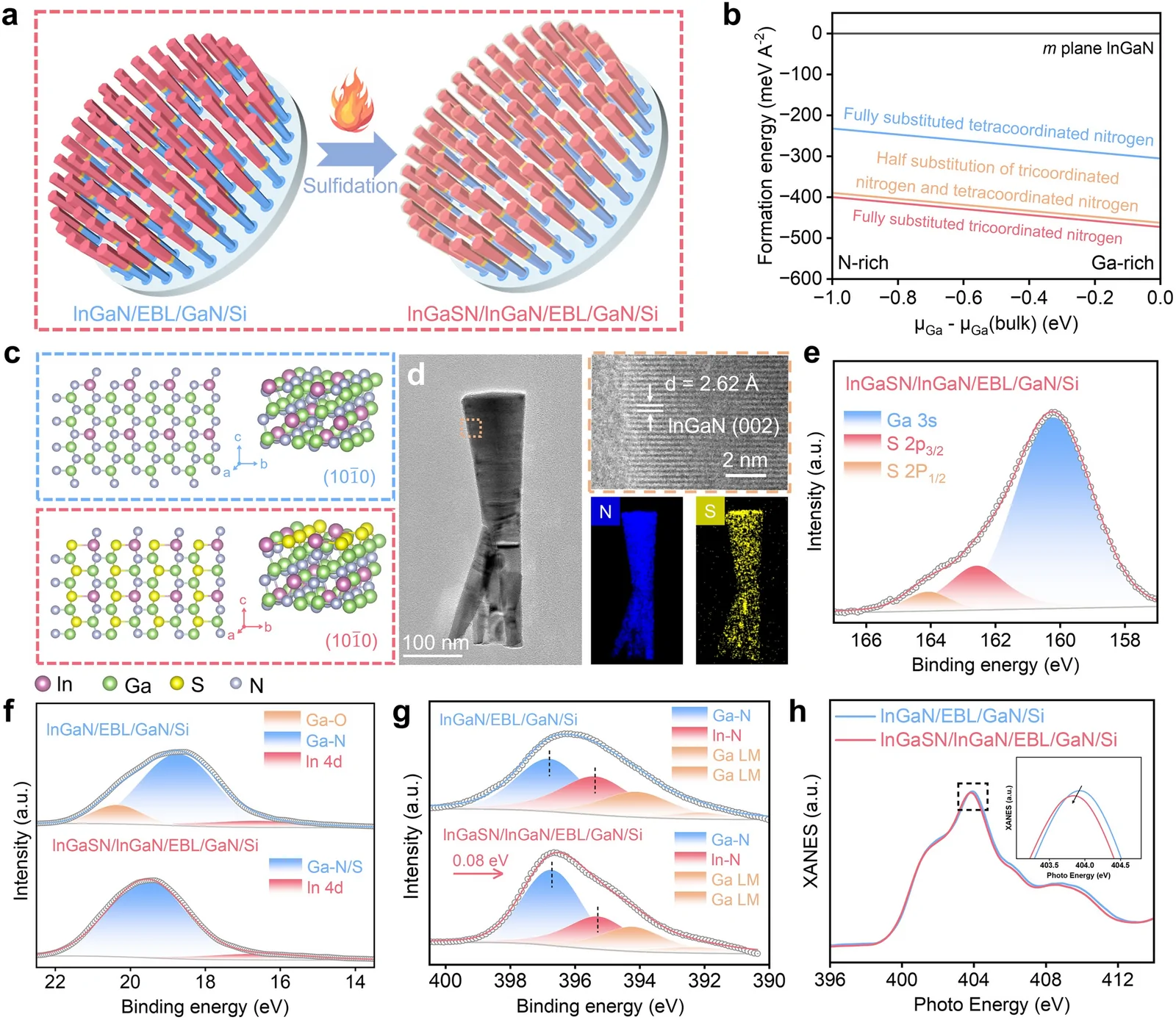
Establishment of the externally optimized microscopic model and electronic structure characterization. **a** Schematic illustration of the InGaSN/InGaN/EBL/GaN/Si and InGaN/EBL/GaN/Si models. **b** Calculated formation energy of m-plane InGaN surfaces with different sulfur configurations under anion- and cation-rich limits. **c** Crystal structure illustration of InGaN/EBL/GaN/Si (blue box) and InGaSN/InGaN/EBL/GaN/Si (red box). **d** TEM image of InGaSN/InGaN/EBL/GaN/Si with the HRTEM image at the position indicated by the orange box, along with EDS elemental mapping of sulfur and nitrogen. **e** High-resolution S 2*p* XPS spectrum of InGaSN/InGaN/EBL/GaN/Si. **f** High-resolution Ga 3*d* XPS spectra of InGaN/EBL/GaN/Si and InGaSN/InGaN/EBL/GaN/Si. **g** High-resolution N 1*s* XPS spectra of InGaN/EBL/GaN/Si and InGaSN/InGaN/EBL/GaN/Si. **h** N *K*-edge spectra of InGaN/EBL/GaN/Si and InGaSN/InGaN/EBL/GaN/Si

By comparing the performance of InGaN/EBL/GaN/Si photocathodes before and after the introduction of electron acceptors, it becomes clear that although the incorporation of an internal EBL markedly increases the number of photogenerated electrons reaching the p-InGaN surface, the intrinsically inert nature of p-InGaN still severely limits electron transfer between the surface and the electrolyte. This limitation highlights the need to develop strategies that establish a rapid electron transfer pathway from the p-InGaN surface to the solution. Notably, wurtzite-type InGaN possesses electronically tunable surfaces [10], which can be modified through surface doping with metal or nonmetal ions to enhance its electronic properties. Such modifications provide a feasible approach for constructing an efficient electron transfer channel from the p-InGaN surface to the electrolyte. Here, sulfur ions are introduced onto the inert InGaN surface via a simple sulfuration process (Fig. 3a), thereby constructing an InGaSN/InGaN surface structure. (The detailed experimental steps can be found in the Methods section.) To visually present the atomic structure of the InGaSN/InGaN surface, we performed DFT calculations to evaluate the formation energies of several representative sulfur-doped surface models (Fig. S5). As illustrated in Fig. 3b, compared with other sulfide surface models, the formation energy of the sulfur atoms fully superseding the tricoordinated nitrogen atoms on the InGaN surface is the lowest, resulting in a more stable structure that is easier to form. Figure 3c shows the atomic model of the sulfur atoms fully superseding the tricoordinated nitrogen atoms on the InGaN surface and the original InGaN surface atomic structure. The microstructure of the sulfide nanowires was subsequently characterized via electron microscopy. SEM analysis indicated that there was no significant change in the microstructure of the nanowires after sulfuration (Fig. S6). Additionally, the TEM and HRTEM images revealed that no obvious sulfide particles formed on the surface of the InGaN NWs after sulfurization, and the nanowires still exhibited clear lattice fringes, indicating that their high crystallinity remained unchanged (Fig. 3d). Further EDS characterization of the sulfide nanowires revealed that sulfur and nitrogen were uniformly distributed along the entire length of the nanowires, with no noticeable elemental aggregation observed (Fig. 3d), thereby confirming the successful incorporation of sulfur.

To gain a deeper understanding of the surface chemical state and electronic interactions of the InGaN nanowires after sulfuration, X-ray photoelectron spectroscopy (XPS) and surface-sensitive soft X-ray absorption spectroscopy (sXAS) were conducted. From the S 2*p* spectrum of InGaSN/InGaN/EBL/GaN/Si (Fig. 3e), the characteristic peaks at 162.58 and 164.09 eV are assigned to S 2*p*_3/2_ and S 2*p*_1/2_, respectively, while the peak at 160.21 eV corresponds to Ga 3*s*, further confirming the successful incorporation of sulfur [37–39]. The Ga 3*d* spectra shown in Fig. 3f further indicate that after sulfurization, the Ga-N characteristic peak initially located at 18.72 eV [40, 41] shifts significantly to 19.55 eV, while the original Ga-O peak disappears. The disappearance of the Ga-O peak may be attributed to the effect of a reducing atmosphere on the slightly oxidized surface. Additionally, as shown in Fig. 3g, after surface sulfuration treatment, the N 1*s* spectrum of the nanowires remained stable, with the presence of In-N and Ga-N bonds, indicating that the sulfur atoms did not completely replace all the nitrogen atoms on the InGaN surface. Moreover, a negative shift in the energy position of the N 1*s* XPS spectrum was observed, suggesting electron accumulation around the nitrogen sites. To further investigate this phenomenon, surface-sensitive X-ray absorption spectroscopy was performed. The four peaks in Fig. 3h are attributed to two distinct types of N-metal bonds in the wurtzite structure of III-group nitrides. Compared with InGaN/EBL/GaN/Si, the nitrogen *K*-edge spectrum of InGaSN/InGaN/EBL/GaN/Si exhibited a significant reduction in the intensity of the characteristic peaks, along with a noticeable blueshift, further confirming the formation of an electron-rich region around the nitrogen sites [8, 42, 43].

### PEC Water Splitting Performance after Surface Sulfuration

**Fig. 4:**
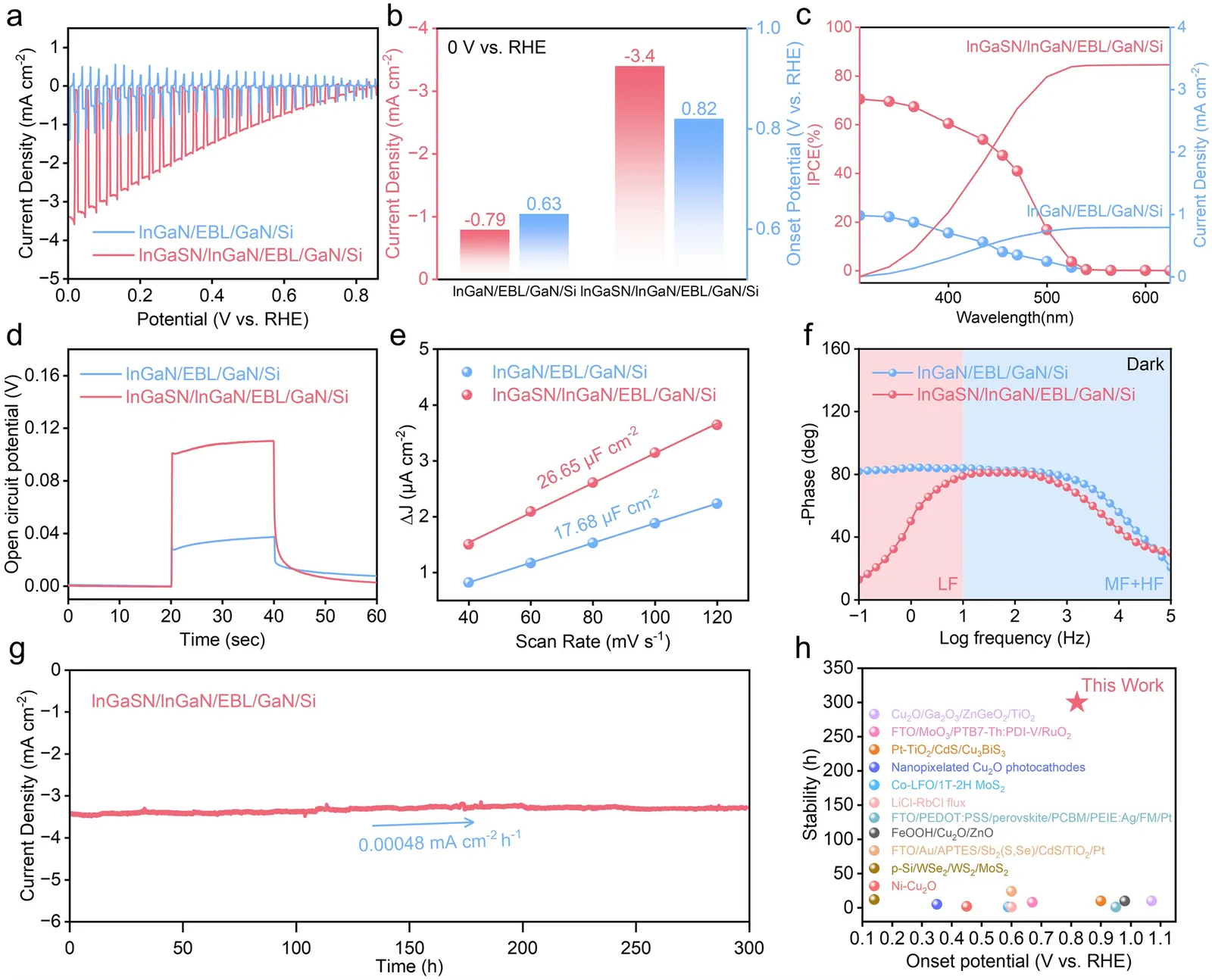
Characterization of photoelectrochemical hydrogen evolution performance following internal optimization. **a** Chopped J–V curves of InGaN/EBL/GaN/Si and InGaSN/InGaN/EBL/GaN/Si. **b** Current density and onset potential of InGaN/EBL/GaN/Si and InGaSN/InGaN/EBL/GaN/Si at 0 V vs. RHE. **c** IPCE curves of InGaN/EBL/GaN/Si and InGaSN/InGaN/EBL/GaN/Si. **d** OCP of InGaN/EBL/GaN/Si and InGaSN/InGaN/EBL/GaN/Si under light and dark conditions. **e** ECSA comparison of InGaN/EBL/GaN/Si and InGaSN/InGaN/EBL/GaN/Si estimated from C_dl_ values. **f** Bode plots of InGaN/EBL/GaN/Si and InGaSN/InGaN/EBL/GaN/Si measured in the dark. **g** Long-term stability evaluation of InGaSN/InGaN/EBL/GaN/Si under 0 V vs RHE in 0.5 M H_2_SO_4_ solution with AM 1.5 G illumination. **h** Comparison of InGaSN/InGaN/EBL/GaN/Si with previously reported advanced photoelectrodes for the hydrogen evolution reaction in terms of stability and onset potential (Table S2)

To further examine the effect of the electron-rich surface formed after sulfurization on the PEC hydrogen evolution activity of InGaSN/InGaN/EBL/GaN/Si, the device was evaluated in a standard three-electrode system using 0.5 M H_2_SO_4_ as the electrolyte. Figure 4a presents the chopped J–V curves of InGaN/EBL/GaN/Si and InGaSN/InGaN/EBL/GaN/Si. Compared with InGaN/EBL/GaN/Si, the sulfurized surface exhibited markedly enhanced PEC hydrogen evolution activity. As shown in Fig. 4b, the photocurrent density at 0 V vs. RHE increases from -0.79 to -3.40 mA cm^−2^, which is further confirmed by the analysis of the i–t curves of the two samples at 0 V vs. RHE (Fig. S7). In addition, the onset potential (V_on_) increased from 0.63 V vs. RHE to 0.82 V vs. RHE. Here, we define the onset potential as the potential value corresponding to the intercept between the extrapolated tangent lines of the J–V curves measured during illumination (AM 1.5 G, 100 mW cm^−2^) and in the dark (Fig. S8) [44]. Subsequently, the applied bias photon-to-current efficiency (ABPE) curve was derived from the LSV data. As shown in Fig. S9, the maximum ABPE of InGaN/EBL/GaN/Si was only 0.070% at 0.183 V vs. RHE, whereas that of InGaSN/InGaN/EBL/GaN/Si reached 0.611% at 0.374 V vs. RHE—an improvement of approximately 8.73-fold. Furthermore, Fig. 4c shows the IPCE spectra of InGaN/EBL/GaN/Si and InGaSN/InGaN/EBL/GaN/Si measured at 0 V vs. RHE. Both samples exhibited similar spectral trends, but the IPCE values of InGaSN/InGaN/EBL/GaN/Si were consistently higher across all wavelengths. Integration of the IPCE curve with the AM 1.5G simulated solar spectrum yielded a photocurrent density consistent with the measured value, confirming that the photocurrent originates from photon-induced electron excitation. Subsequently, the InGaN/GaN/Si structure was subjected to the sulfurization treatment, and its performance was evaluated. As shown in Fig. S10, the PEC hydrogen evolution activity of InGaSN/InGaN/GaN/Si was markedly improved compared with that of InGaN/GaN/Si, although it remained inferior to the performance of InGaSN/InGaN/EBL/GaN/Si. These findings further confirm the effectiveness of the dual-electron extraction strategy based on internal–external synergy.

The open-circuit potential (OCP) of both photoelectrodes was subsequently measured, as shown in Fig. 4d. The ΔOCP of InGaSN/InGaN/EBL/GaN/Si reached 0.11 V, considerably higher than the 0.037 V measured for InGaN/EBL/GaN/Si, thereby indicating that surface sulfurization enhances the driving force for photogenerated electrons to reach the hydrogen evolution reaction (HER) interface. The double-layer capacitance (C_dl_) was subsequently determined via cyclic voltammetry (CV) to evaluate the electrochemical surface area (ECSA) of the photoelectrodes (Fig. S11). As shown in Fig. 4e, the C_dl_ value of InGaSN/InGaN/EBL/GaN/Si (26.65 μF cm^−2^) exceeded that of InGaN/EBL/GaN/Si (17.68 μF cm^−2^), confirming that sulfurization of the InGaN surface introduced additional active sites for PEC hydrogen evolution and provided a larger effective reaction area. Moreover, charge transfer at the surface/electrolyte interface before and after sulfurization was analyzed via Bode plots under dark conditions, with particular attention given to phase-angle variations in the LF region. As shown in Fig. 4f, the phase angle of InGaSN/InGaN/EBL/GaN/Si was significantly smaller than that of InGaN/EBL/GaN/Si, demonstrating that surface sulfurization effectively reduces the interfacial charge transfer resistance and thereby facilitates electron transfer. Finally, as shown in Fig. 4g, the long-term stability of the InGaSN/InGaN/EBL/GaN/Si photoelectrode was evaluated. After approximately 300 h of operation, the hydrogen evolution performance of the single-junction InGaSN/InGaN/EBL/GaN/Si photocathode without any protective overlayer decreased at a slow rate of 0.00048 mA cm^−2^h^−1^, indicating excellent stability. Subsequently, SEM, TEM, and XPS analyses were conducted on the InGaSN/InGaN/EBL/GaN/Si after the stability test (Figs. S12 and S13). In summary, by employing the dual-electron extraction strategy based on internal–external synergy, the photocurrent density of the single-junction p-InGaN photocathode at 0 V vs. RHE increased from −0.09 to −3.40 mA cm^−2^ (Table S1), achieving a remarkable 37.8-fold enhancement. Moreover, as shown in Fig. 4h, when compared with several state-of-the-art photocathodes developed in recent years, the final InGaSN/InGaN/EBL/GaN/Si photocathode exhibits relatively good stability and onset potential. These results highlight the effectiveness of our dual-electron extraction strategy based on internal–external synergy.

### Performance Regulatory Mechanism Induced by the InGaSN Layer

**Fig. 5:**
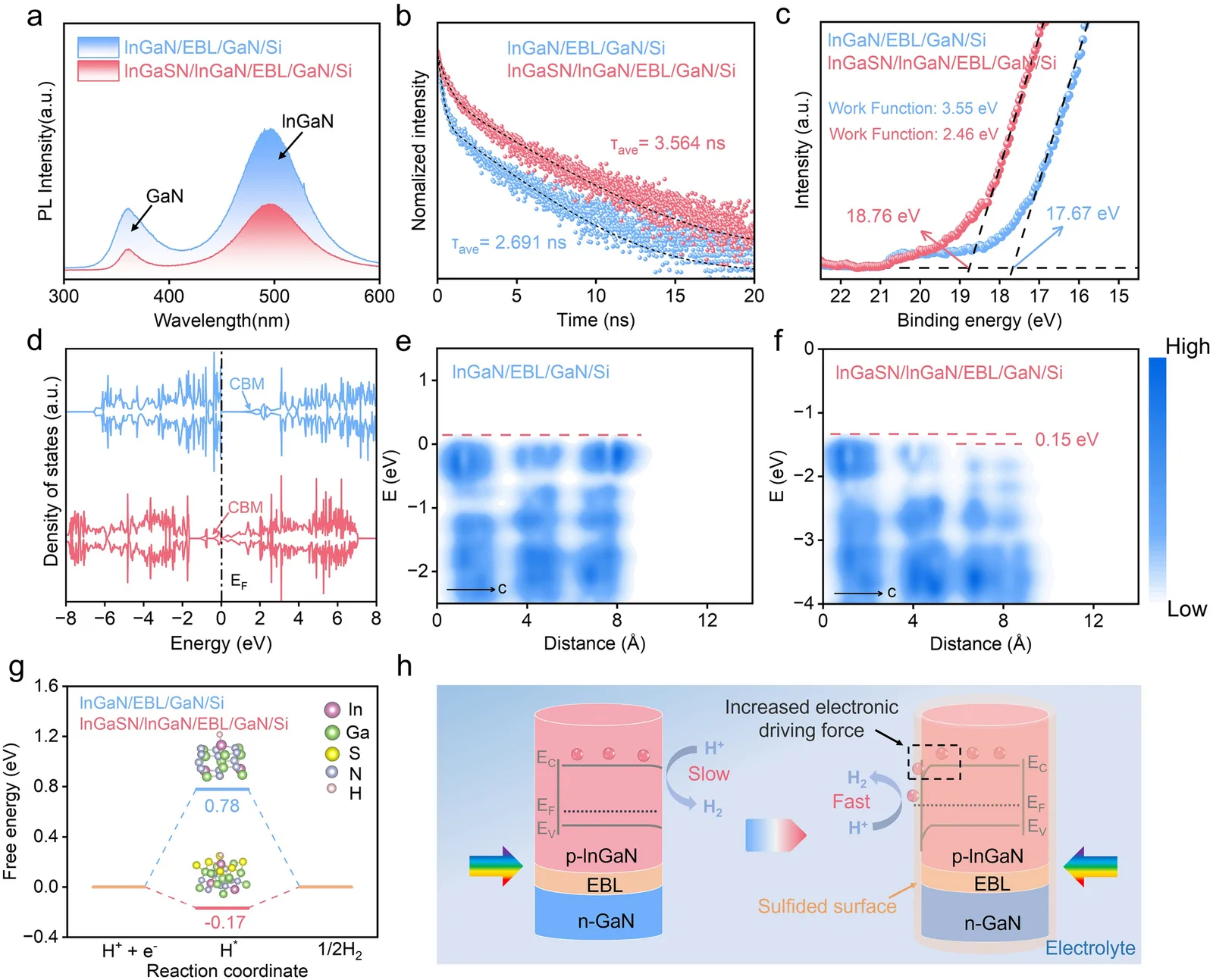
Revealing the reasons for external optimization. **a** PL spectra of InGaN/EBL/GaN/Si and InGaSN/InGaN/EBL/GaN/Si at an excitation wavelength of 266 nm. **b** TRPL spectra of InGaN/EBL/GaN/Si and InGaSN/InGaN/EBL/GaN/Si. **c** Work functions of InGaN/EBL/GaN/Si and InGaSN/InGaN/EBL/GaN/Si calculated by UPS. **d** DOS of InGaN/EBL/GaN/Si (blue curve) and InGaSN/InGaN/EBL/GaN/Si (red curve). **e, f** LDOS color maps for InGaN/EBL/GaN/Si and InGaSN/InGaN/EBL/GaN/Si. **g** Gibbs free energy of hydrogen adsorption on InGaN/EBL/GaN/Si and InGaSN/InGaN/EBL/GaN/Si during the HER process. **h** Mechanistic illustration of enhanced photoelectrochemical hydrogen evolution performance after surface sulfuration (red spheres represent electrons)

On the basis of the performance characterization results, the PEC hydrogen evolution efficiency of single-junction p-InGaN was markedly enhanced after surface sulfurization. To probe the underlying mechanisms, steady-state photoluminescence (PL) and time-resolved photoluminescence (TRPL) measurements were conducted to examine changes in carrier dynamics induced by sulfurization of the p-InGaN surface. Analysis of the PL spectra (Fig. 5a) reveals that sulfurization significantly suppresses the recombination of photogenerated electron–hole pairs [45, 46]. Furthermore, the TRPL results (Fig. 5b) show that the carrier lifetime of InGaSN/InGaN/EBL/GaN/Si (3.564 ns) is longer than that of InGaN/EBL/GaN/Si (2.691 ns), confirming that sulfurization enhances effective carrier separation [47]. The in situ irradiated XPS (ISI-XPS) results (Fig. S14) show that the S *2p* energy level shifts negatively after illumination, indicating surface electron enrichment, which is favorable for the hydrogen evolution reaction. Subsequently, ultraviolet photoelectron spectroscopy (UPS) revealed that surface sulfurization treatment led to a significant reduction in the work function (Fig. 5c), decreasing from 3.55 eV for InGaN/EBL/GaN/Si to 2.46 eV for InGaSN/InGaN/EBL/GaN/Si, indicating that the surface sulfurization treatment caused a pronounced upward shift of the Fermi level at the photoelectrode surface.

To further investigate the origin of the upward shift of the Fermi level, we performed density functional theory (DFT) calculations based on the theoretical model shown in Fig. 3c (see Methods). First, by analyzing the calculated electrostatic potential distribution and density of states (DOS) plots (Figs. S15 and 5d), we confirmed that the work function of surface InGaSN is significantly lower than that of bulk InGaN, which is consistent with the UPS experimental results. This phenomenon may occur because sulfur atoms have one more valence electron than the substituted nitrogen atoms. Thus, it can be simply regarded as effective n-type doping, which increases the surface electron filling level and consequently shifts the Fermi level upward [48–50]. Consequently, as shown in Fig. S16, when surface InGaSN comes into contact with bulk InGaN, electrons in surface InGaSN flow into bulk InGaN to achieve Fermi level alignment, resulting in pronounced downward band bending in bulk InGaN [51]. Furthermore, we calculated the local density of states (LDOS) (Fig. 5e, f), and the plane-averaged DOS further revealed changes in the local electronic structure. Based on LDOS, we confirmed that sulfur doping induces pronounced downward band bending near the surface. To quantify this effect, we defined the energy difference (Δ*E*) between the maximum valence band edge (VBM) of the bulk material and the surface VBM position [43]. The results show that sulfur doping increases Δ*E* from 0 to 0.15 eV. In addition, the η_sep_ of the InGaSN/InGaN/EBL/GaN/Si nanowires increases markedly (Fig. S17), further supporting improved carrier separation and more efficient interfacial charge transport after sulfurization. Finally, the reaction activity is evaluated by calculating the adsorption energy. For the HER, the reaction activity can be estimated by the Gibbs free energy of hydrogen adsorption (|ΔG_H*_|). According to Sabatier’s principle, the optimal hydrogen adsorption free energy should be maintained at around 0 eV, which is conducive to both the adsorption of H* and the desorption of H_2_ [8, 52]. In Fig. 5g, the ΔG_H*_ for InGaN/EBL/GaN/Si is 0.78 eV, indicating that it is difficult to adsorb H*, which hinders the HER. In contrast, InGaSN/InGaN/EBL/GaN/Si corresponds to a ΔG_H*_ of −0.17 eV, which is closer to 0 eV, indicating weaker hydrogen adsorption and facile desorption of H_2_, resulting in better HER activity and faster hydrogen evolution reaction kinetics. Meanwhile, the smaller Tafel slope and larger exchange current density (j_0_) after surface sulfurization (Fig. S18) further validate this conclusion. In summary, as shown in Fig. 5h, compared with the original inert InGaN surface, the sulfur-ion-doped surface not only improves the band structure of InGaN, thereby facilitating the flow of photogenerated electrons toward the HER reaction interface, but also exhibits superior hydrogen adsorption energy, thus enhancing the HER efficiency.

## Conclusions

In summary, we present a scalable dual-electron extraction strategy with internal–external synergy that fundamentally enhances photogenerated utilization in single-junction p-InGaN NWs. Internally, an EBL is integrated within the NW to effectively suppress electron backflow toward the substrate, thereby directing photogenerated carriers to the NW/electrolyte interface while simultaneously enabling efficient hole collection into the circuit. Externally, the sulfurized surface achieved through anion doping effectively improves the band structure of InGaN, thereby enhancing the driving force for electron transfer to the electrolyte. Meanwhile, the sulfurized surface optimizes hydrogen adsorption energy, accelerating the hydrogen evolution reaction rate. As a result, the optimized p-InGaN photoelectrode achieves a photocurrent density of −3.40 mA cm^−2^ at 0 V vs. RHE—representing a 37.8-fold increase over that of the pristine device—with an onset potential of 0.82 V vs. RHE. Remarkably, the device sustains efficient hydrogen evolution for more than 300 h without the use of any surface protection layers, underscoring its durability under harsh acidic conditions. The successful implementation of this strategy provides a universal and scalable approach for carrier utilization, offering a new perspective to mitigating the common bottleneck of single-junction III-V photoelectrodes—namely, the inefficient electron extraction caused by internal backflow and interfacial transfer barriers.

## Supplementary Information

Below is the link to the electronic supplementary material.

Supplementary file1 (DOCX 3.80 MB)
