# Supplementary material for: Wafer-Scaled III-Nitrides Nanowire Photocathodes Enabled by Synergistic Dual-Electron Extraction for Efficient Solar-to-Hydrogen Conversion
Source: Nanomicro Lett. 2026 Apr 17;18:337. doi: 10.1007/s40820-026-02186-9 (PMC13090466; doi:10.1007/s40820-026-02186-9)
Supplement: Supplementary file 1 — Supplementary file1 (DOCX 3.80 MB) [file 40820_2026_2186_MOESM1_ESM.docx]

Supporting Information for

**Wafer-Scale Nanowire Photocathodes Enabled by Synergistic Dual-Electron Extraction for Efficient Solar-to-Hydrogen Conversion**

Xudong Yang^#,1^, Yuying Liu^#,2^, Wei Chen^#,1^, Tianle Zhang^#,3^, Wengang Gu^1^, Xin Liu^1^, Yuanmin Luo^1^, ZhiXiang Gao^1^, Yang Li^1^, Menglong Wang^1^, Weiyi Wang^3^, Ran Long^2^, Wei Hu^3^, Jiajie Xu *^,4^ Haiding Sun*^,1^

^1^ iGaN Laboratory, School of Microelectronics, University of Science and Technology of China, Hefei 230029, P. R. China

^2^ National Synchrotron Radiation Laboratory, University of Science and Technology of China, Hefei, 230027, P. R. China

^3^Hefei National Laboratory for Physical Science at the Microscale, Department of Chemical Physics, University of Science and Technology of China, Hefei, 230027, P. R. China

^4^Microbial Development and Metabolic Engineering Laboratory, School of Marine Science, Ningbo University, Ningbo 315211, P. R. China

^#^ Xudong Yang, Yuying Liu, Wei Chen, and Tianle Zhang contributed equally to this work.

***Corresponding authors. E-mail: [xujiajie@nbu.edu.cn](mailto:xujiajie@nbu.edu.cn) (Jiajie Xu), [haiding@ustc.edu.cn](mailto:haiding@ustc.edu.cn) (Haiding Sun)

**Supplementary Figures**


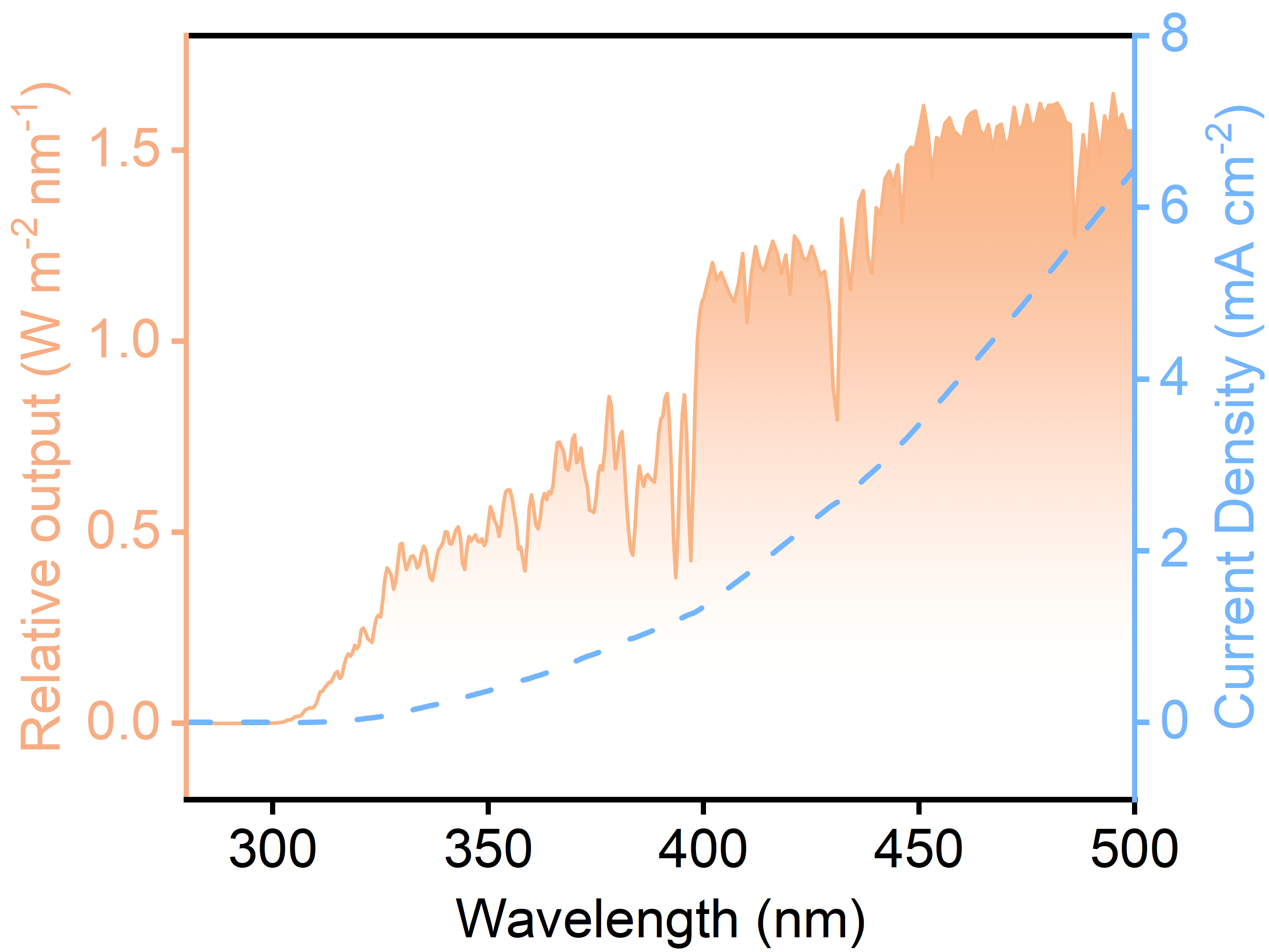


**Fig. S1** The theoretical photocurrent density curve was obtained by integrating the simulated AM 1.5G solar spectrum


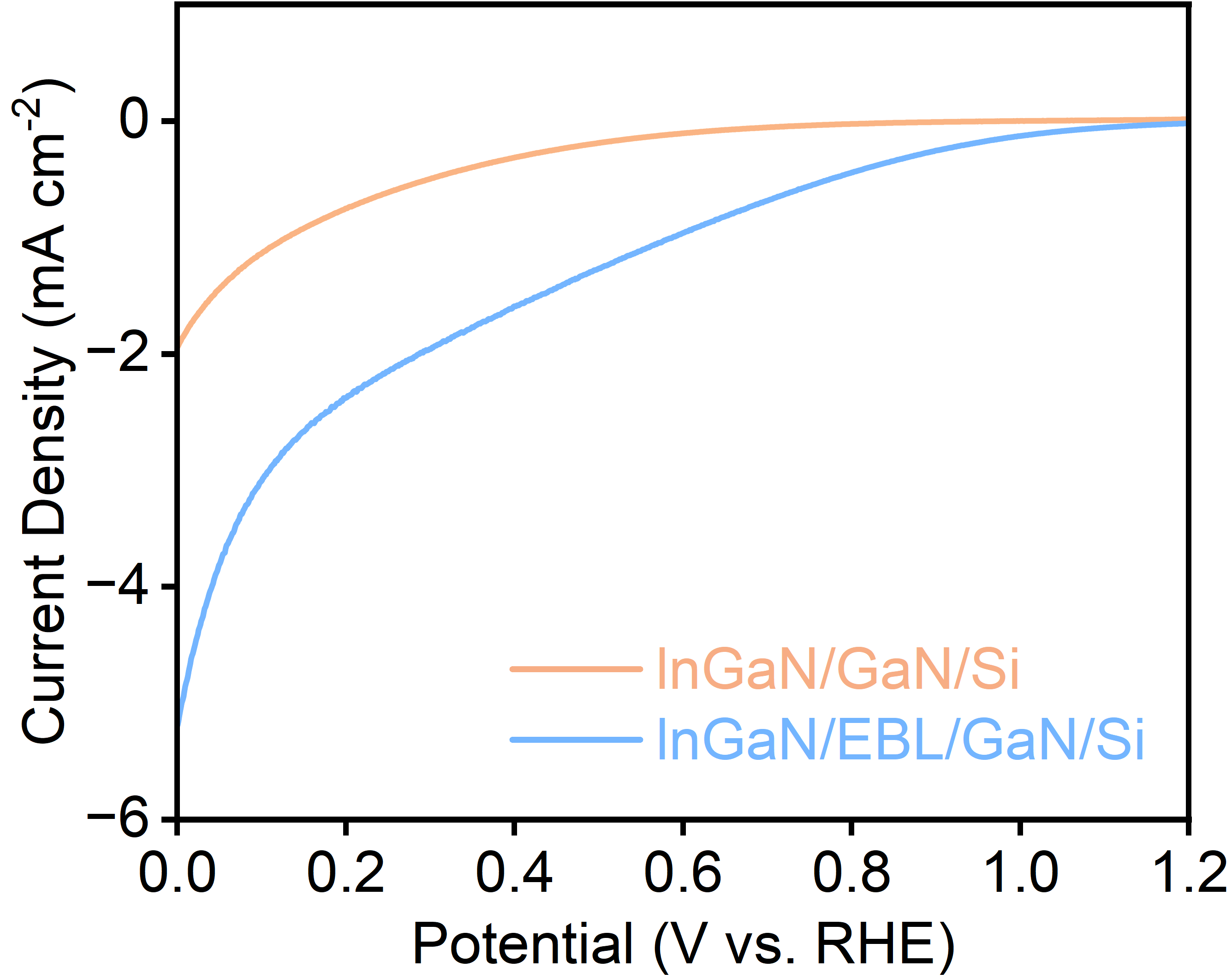


**Fig. S2** LSV curves of InGaN/GaN/Si and InGaN/EBL/GaN/Si photocathodes recorded in 0.5 M Na_2_S_2_O_8_ + 0.5 M H_2_SO_4_ electrolyte under simulated sunlight (AM 1.5G, 100 mW cm^-2^)


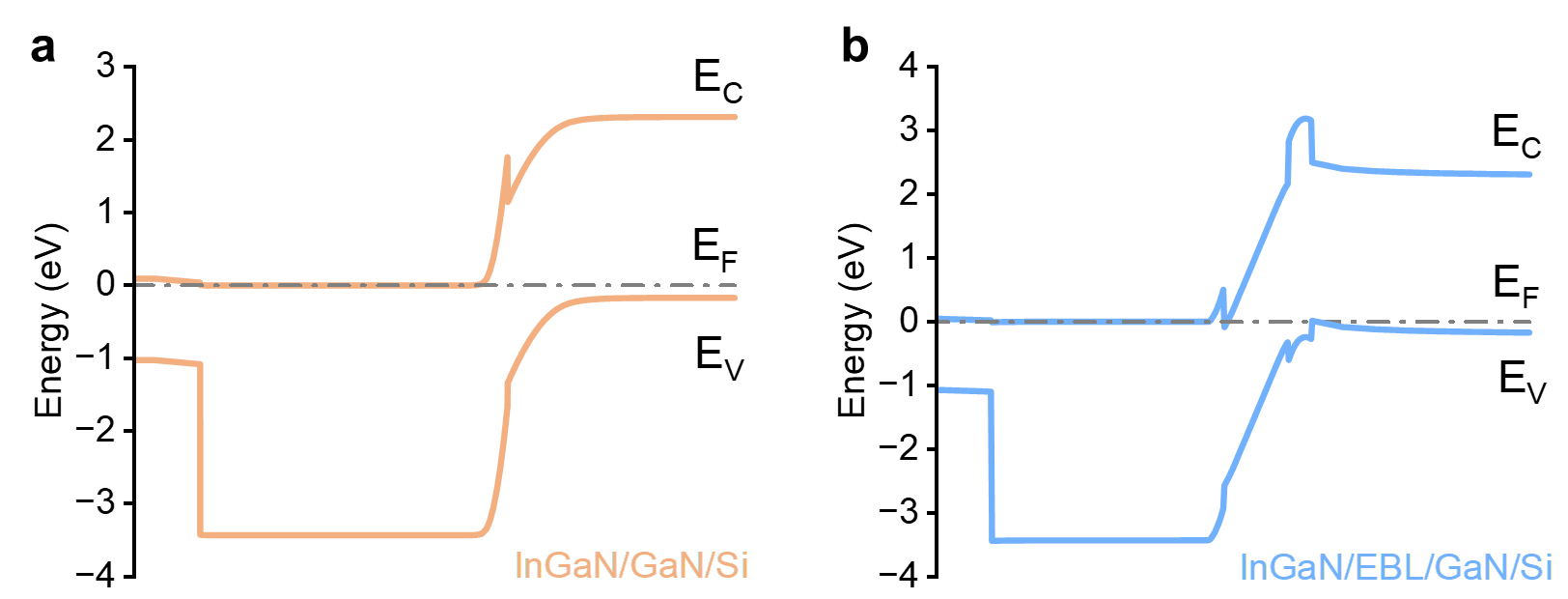


**Fig. S3** Band structures of **a** the InGaN/GaN/Si and **b** the InGaN/EBL/GaN/Si, simulated using APSYS software

The n^++^-GaN/InGaN/p^++^-GaN structure constructed between n-GaN and p-InGaN mainly serves to suppress electron back-injection and leakage. Under heavy doping conditions, the depletion region in the n^++^-GaN/InGaN/p^++^-GaN structure becomes narrowed, enabling holes in p-InGaN to tunnel across the junction via band-to-band tunneling and to be effectively extracted. Meanwhile, the conduction-band offset at the n-GaN interface raises the effective barrier for electron injection/backflow from the top p-InGaN into the underlying n-GaN. Thus, a selective carrier-routing behavior is realized: holes readily cross the junction, whereas electrons are hindered from backflow, leading to enhanced electron availability near the surface.


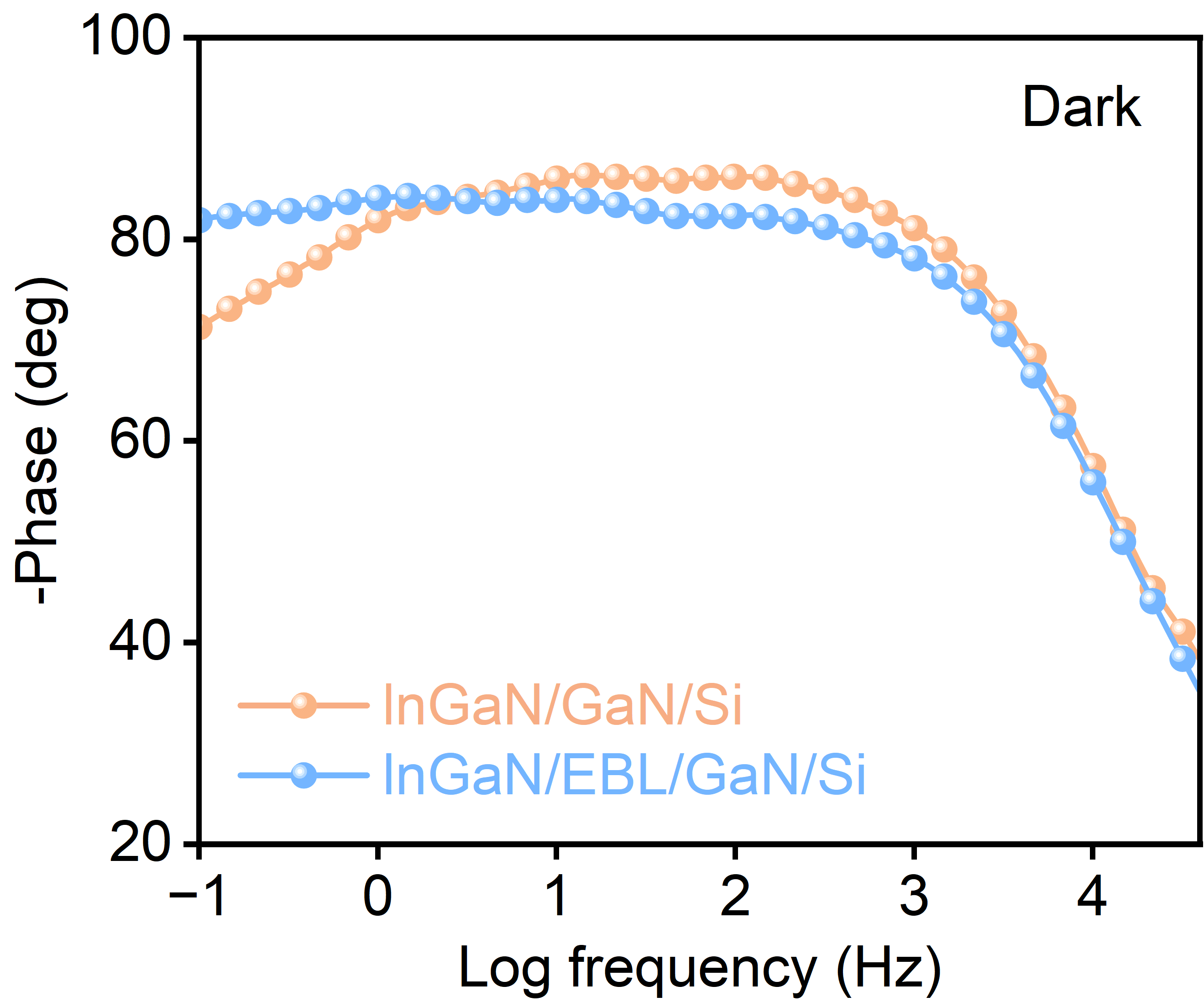


**Fig. S4** Bode plots of InGaN/GaN/Si and InGaN/EBL/GaN/Si photocathodes recorded in the dark


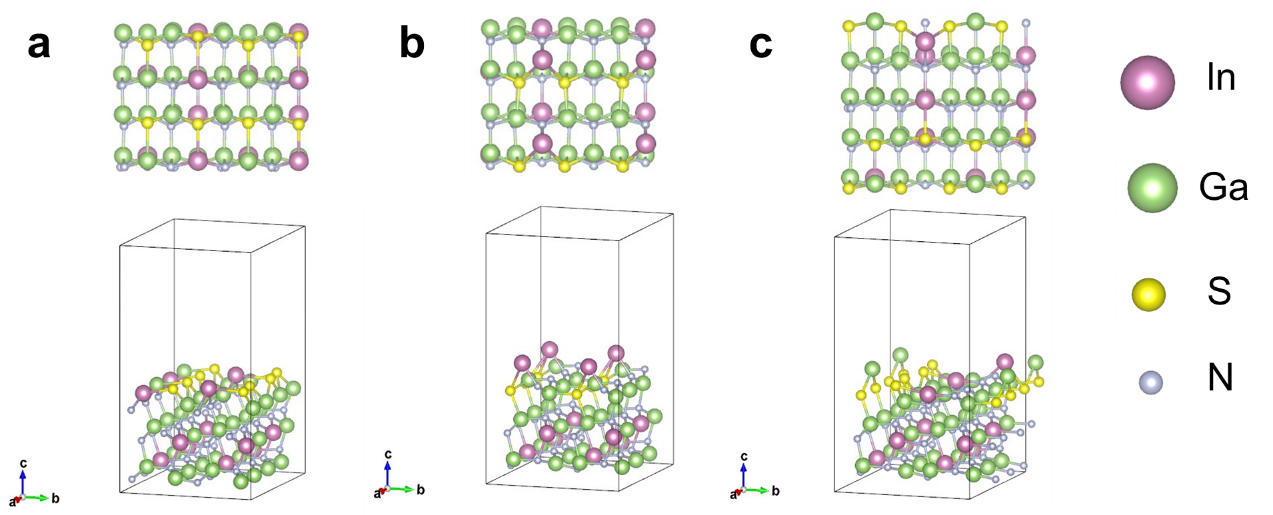


**Fig. S5** Theoretical models of three types of InGaSN surfaces. **a** 50% of the three-coordinated nitrogen atoms on the surface are substituted by sulfur atoms. **b** 50% of the four-coordinated nitrogen atoms on the surface are substituted by sulfur atoms. **c** 25% of the three-coordinated nitrogen atoms and 25% of the four-coordinated nitrogen atoms on the surface are substituted by sulfur atoms. In all cases, the pristine InGaN surface was used as the reference for relative surface formation energy calculations


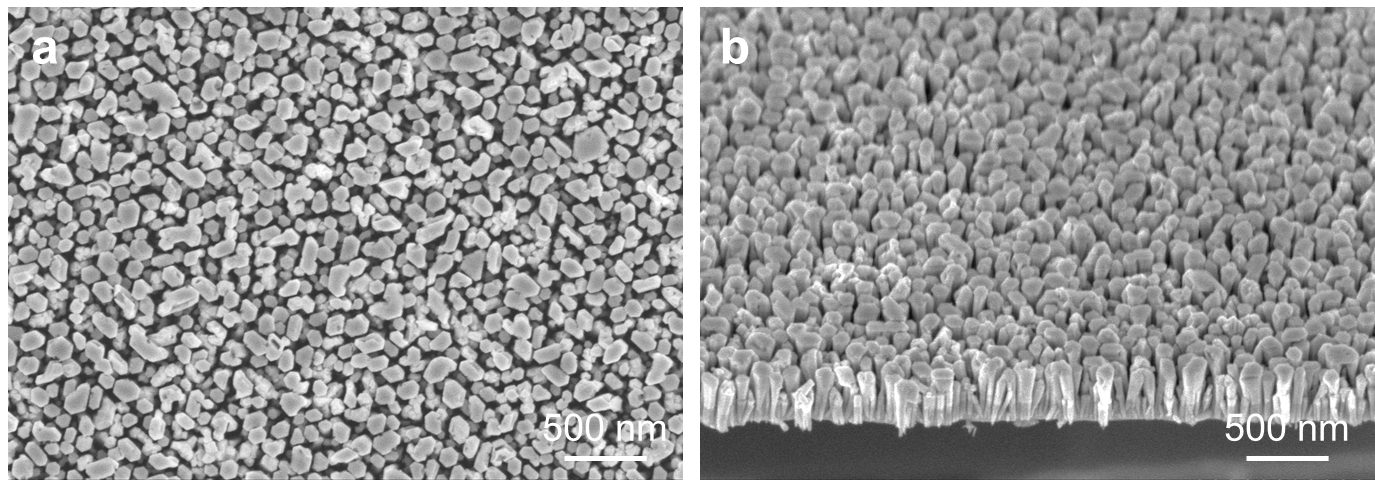


**Fig. S6 a** Top-view and **b** 45° tilted-view SEM images of InGaSN/InGaN/EBL/GaN/Si


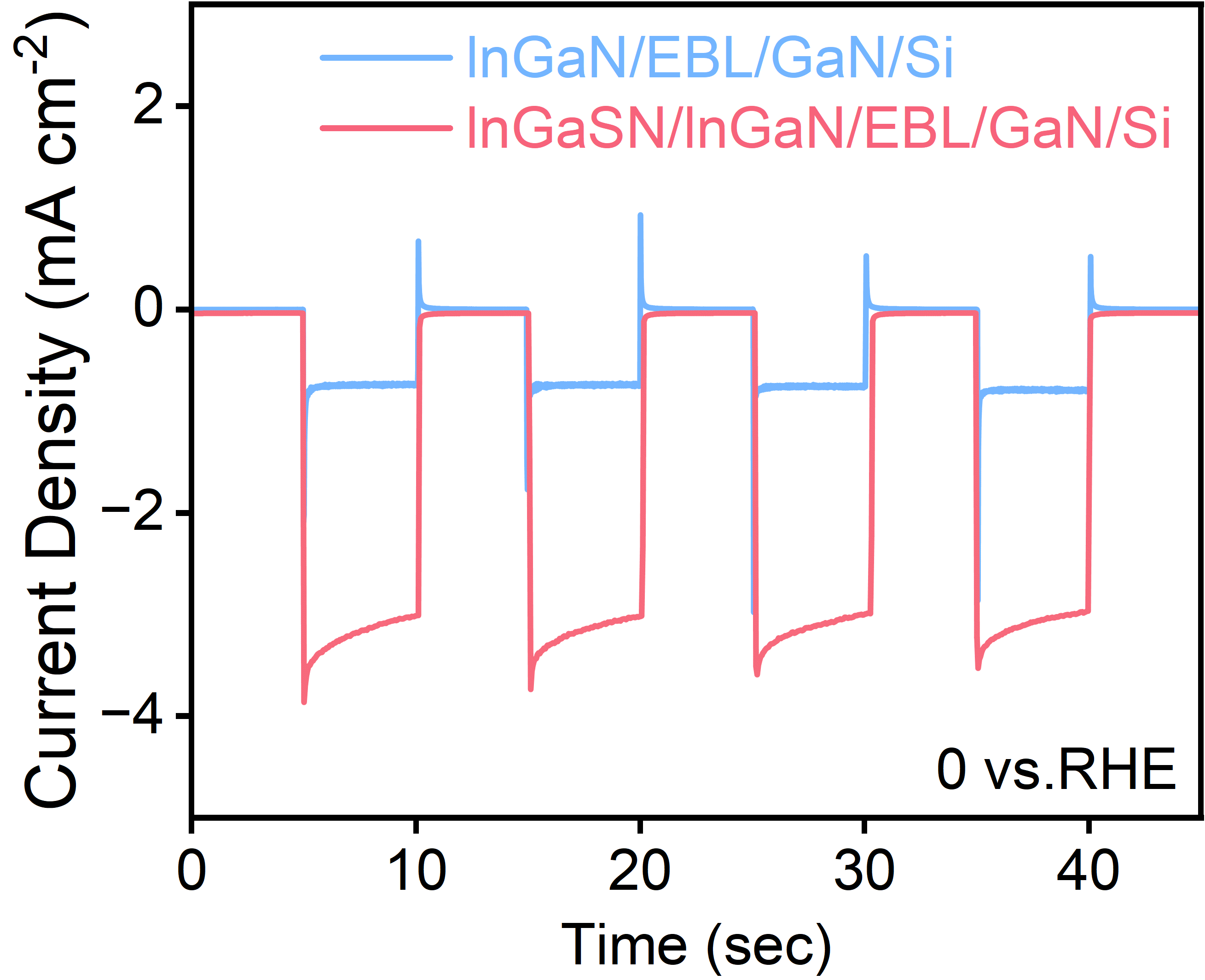


**Fig. S7** I-t curves of InGaN/EBL/GaN/Si and InGaSN/InGaN/EBL/GaN/Si photocathodes measured at 0 V vs. RHE


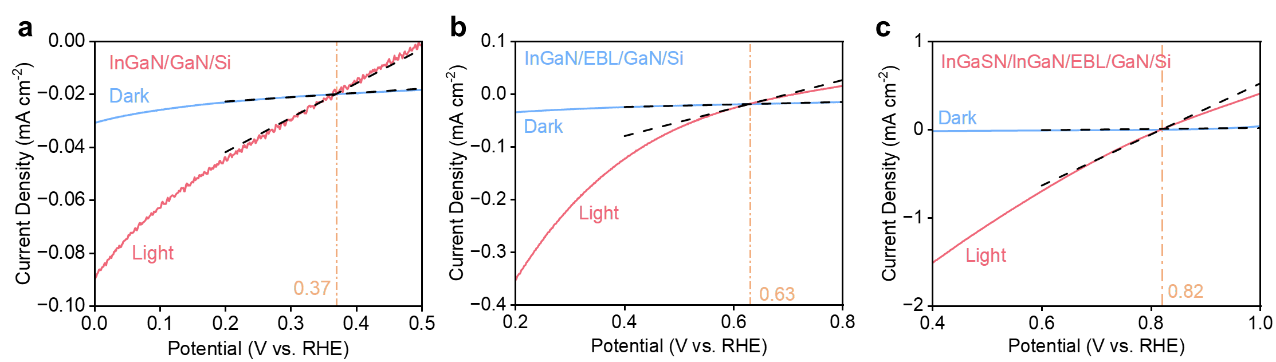


**Fig. S8** Schematic illustration of the onset potential for **a** the InGaN/GaN/Si photocathode, **b** the InGaN/EBL/GaN/Si photocathode, and **c** the InGaSN/InGaN/EBL/GaN/Si photocathode. The onset potential is defined as the potential corresponding to the intercept between the extrapolated tangent lines of the J-V curves measured under illumination (AM 1.5 G, 100 mW cm^-2^) and in the dark


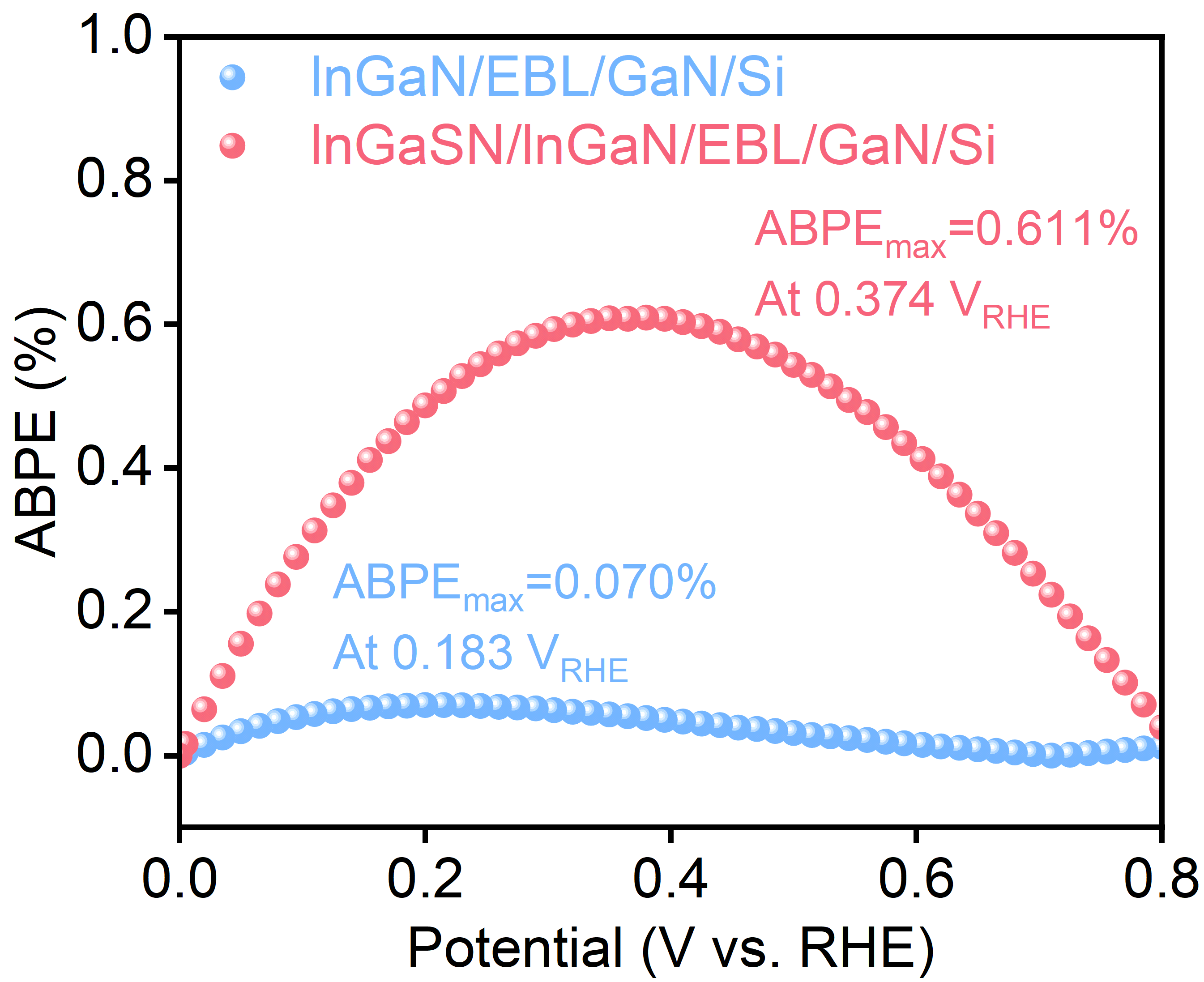


**Fig. S9** ABPE curves of InGaN/EBL/GaN/Si and InGaSN/InGaN/EBL/GaN/Si photocathodes


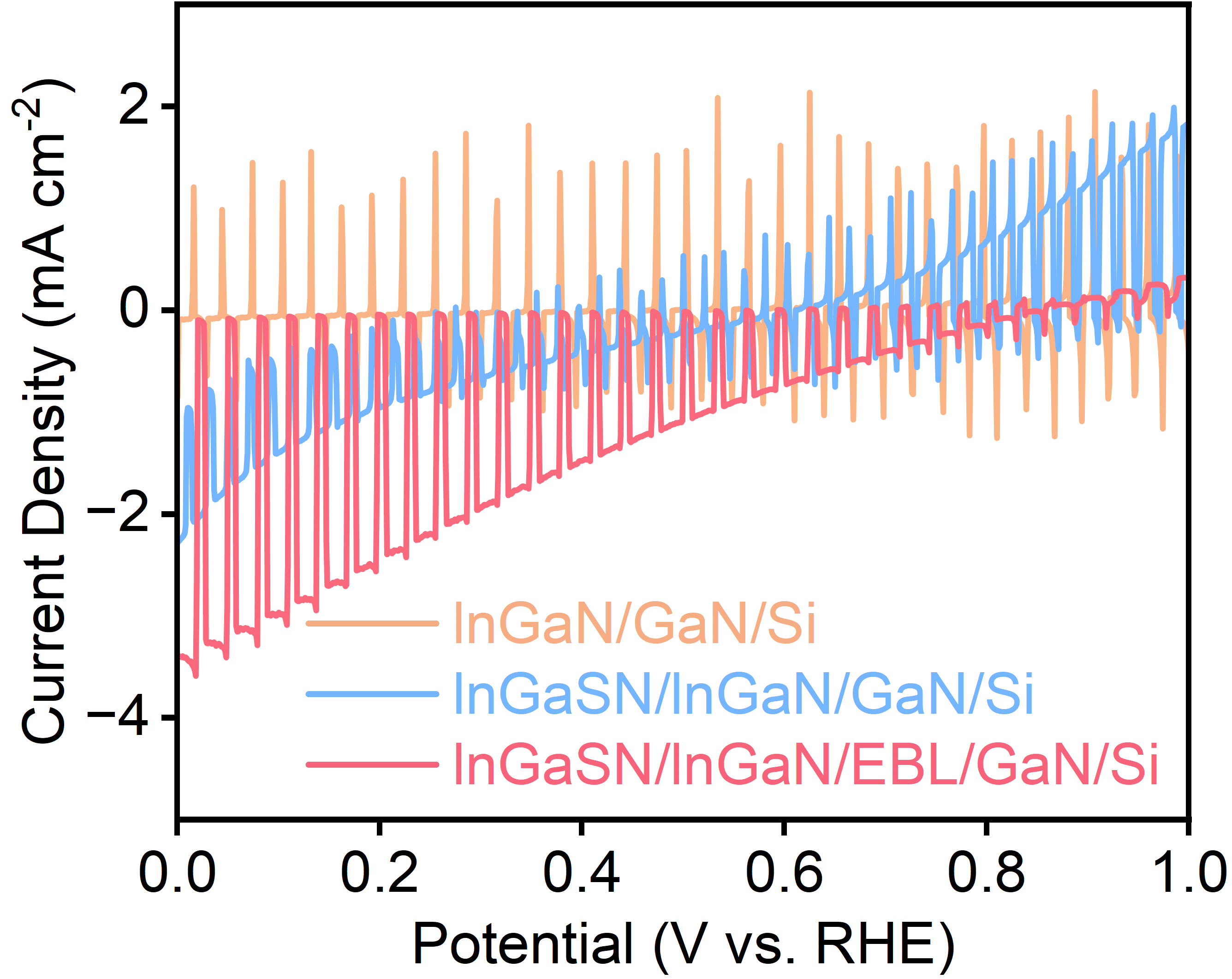


**Fig. S10** Chopped J-V curves of InGaN/GaN/Si, InGaSN/InGaN/GaN/Si, and InGaSN/InGaN/EBL/GaN/Si photocathodes


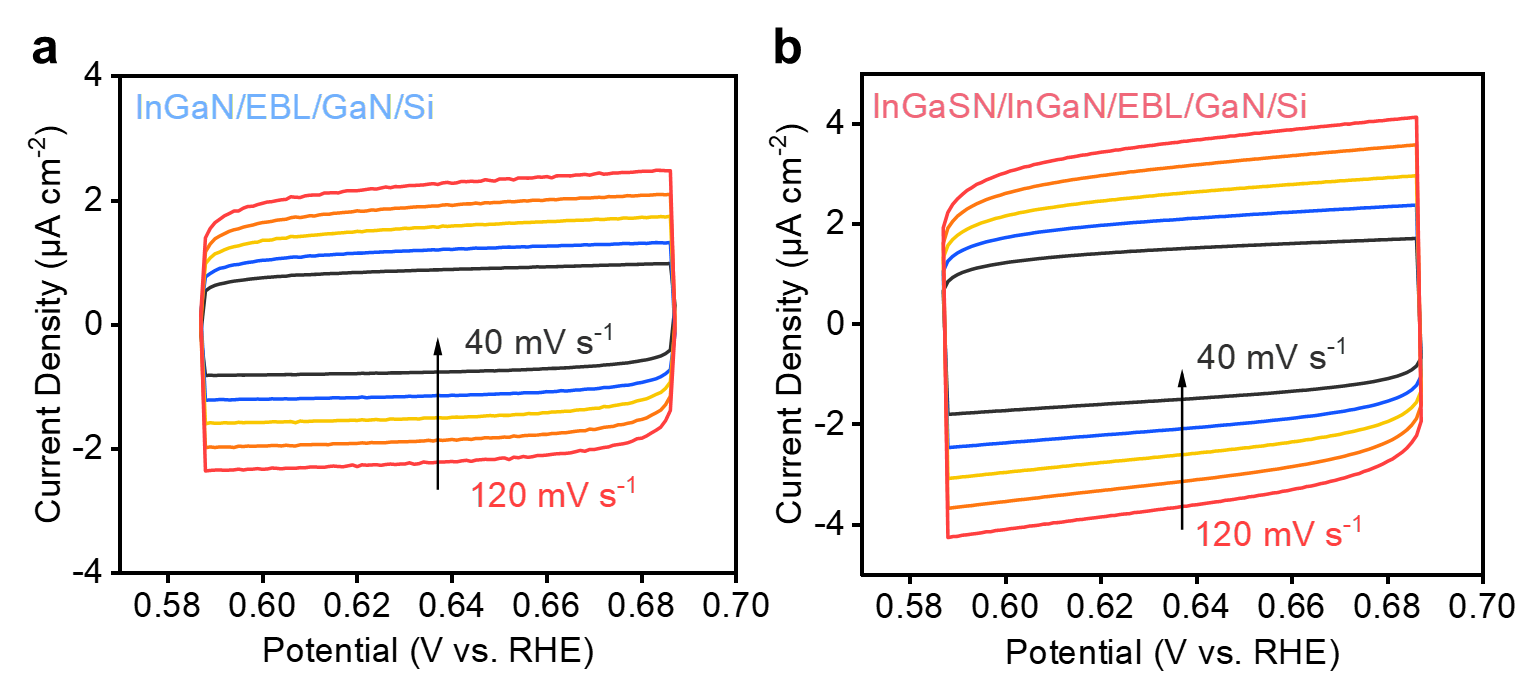


**Fig. S11** Electrochemically active surface area characterization. Cyclic voltammograms of **a** the InGaN/EBL/GaN/Si photocathode and **b** the InGaSN/InGaN/EBL/GaN/Si photocathode


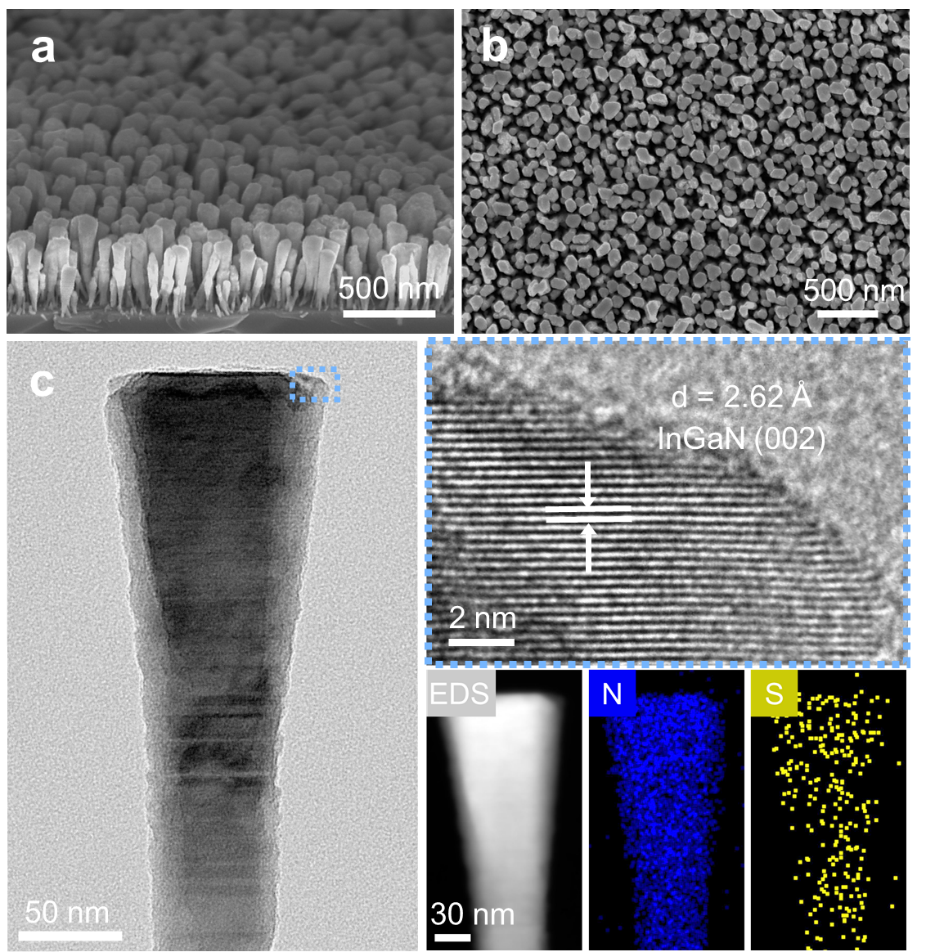


**Fig. S12** **a** 45° tilted‑view and **b** top-view SEM images of InGaSN/InGaN/EBL/GaN/Si after 300 h stability testing. **c** TEM image of InGaSN/InGaN/EBL/GaN/Si after 300 h stability testing with the HRTEM image at the position indicated by the blue box, along with EDS elemental mapping of sulfur and nitrogen.

The SEM images indicate that the overall morphology of the sample remains unchanged, with no signs of degradation. TEM analysis further confirms that its microstructure is intact, and HRTEM reveals lattice fringes corresponding to the (002) plane of InGaN with a spacing of d = 2.62 Å, demonstrating that the high crystallinity is unaffected. Meanwhile, the EDS mapping results show that sulfur remains uniformly distributed after the reaction, with no evidence of local enrichment or obvious phase separation, supporting the sustained presence of the InGaSN surface layer after long-term operation.


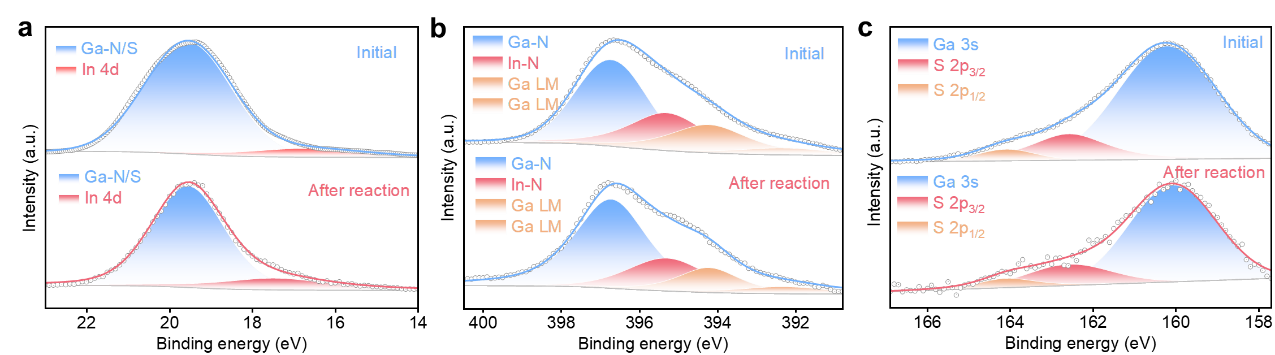


**Fig. S13** **a** High-resolution Ga 3d spectra of InGaSN/InGaN/EBL/GaN/Si before and after long-term stability testing. **b** High-resolution N 1s spectra of InGaSN/InGaN/EBL/GaN/Si before and after long-term stability testing. **c** High-resolution S 2p spectra of InGaSN/InGaN/EBL/GaN/Si before and after long-term stability testing.

XPS analysis further indicates that the peak positions and spectral features of Ga 3d, N 1s, and S 2p remain consistent before and after the reaction, with no significant differences. This confirms that the sulfur-related surface species are well preserved, and no apparent surface oxidation or chemical state changes occur, thereby verifying the chemical stability of the InGaSN surface layer.

**
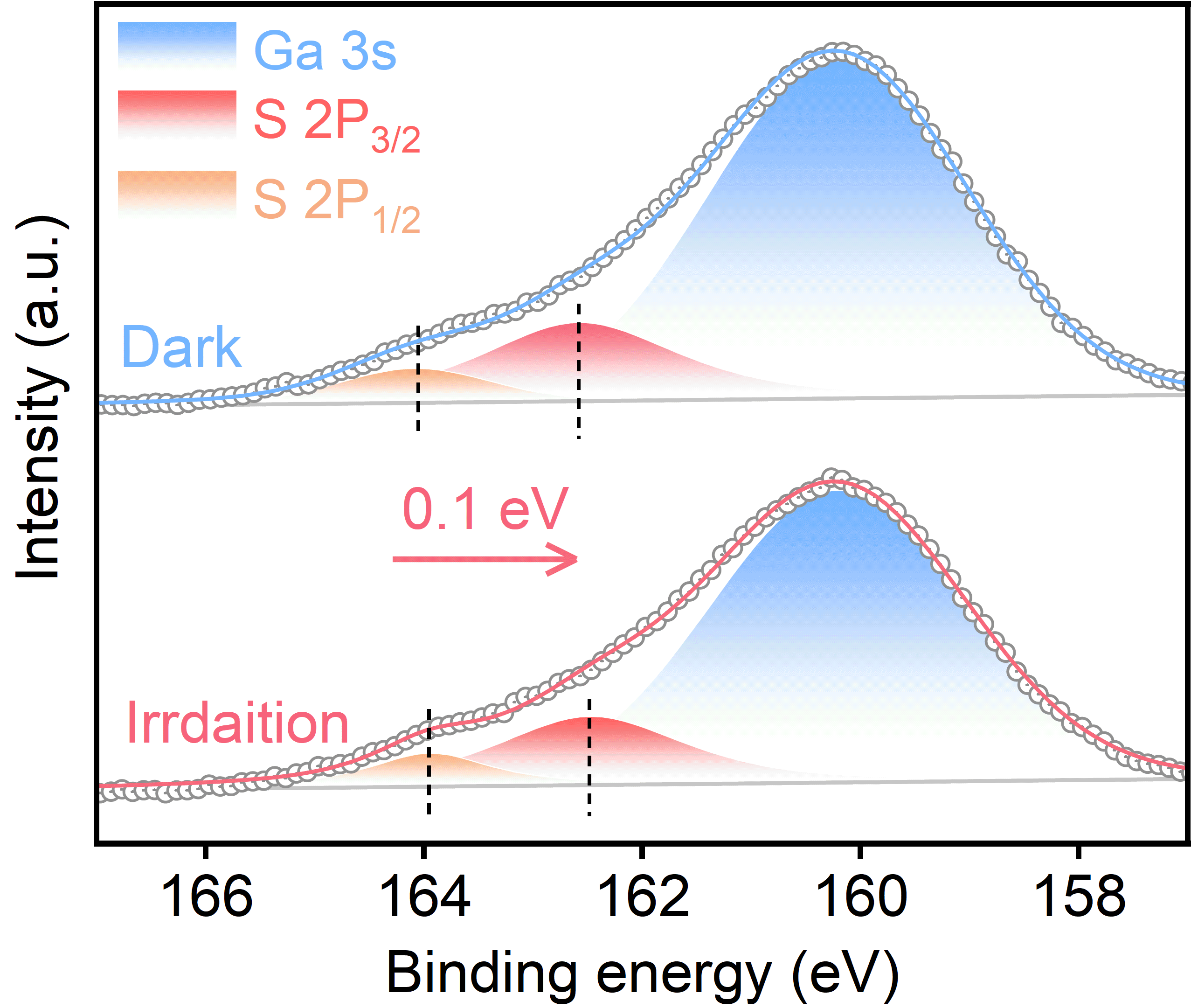
**

**Fig. S14** ISI-XPS spectra of S 2p for the InGaSN/InGaN/EBL/GaN/Si photocathode.

**
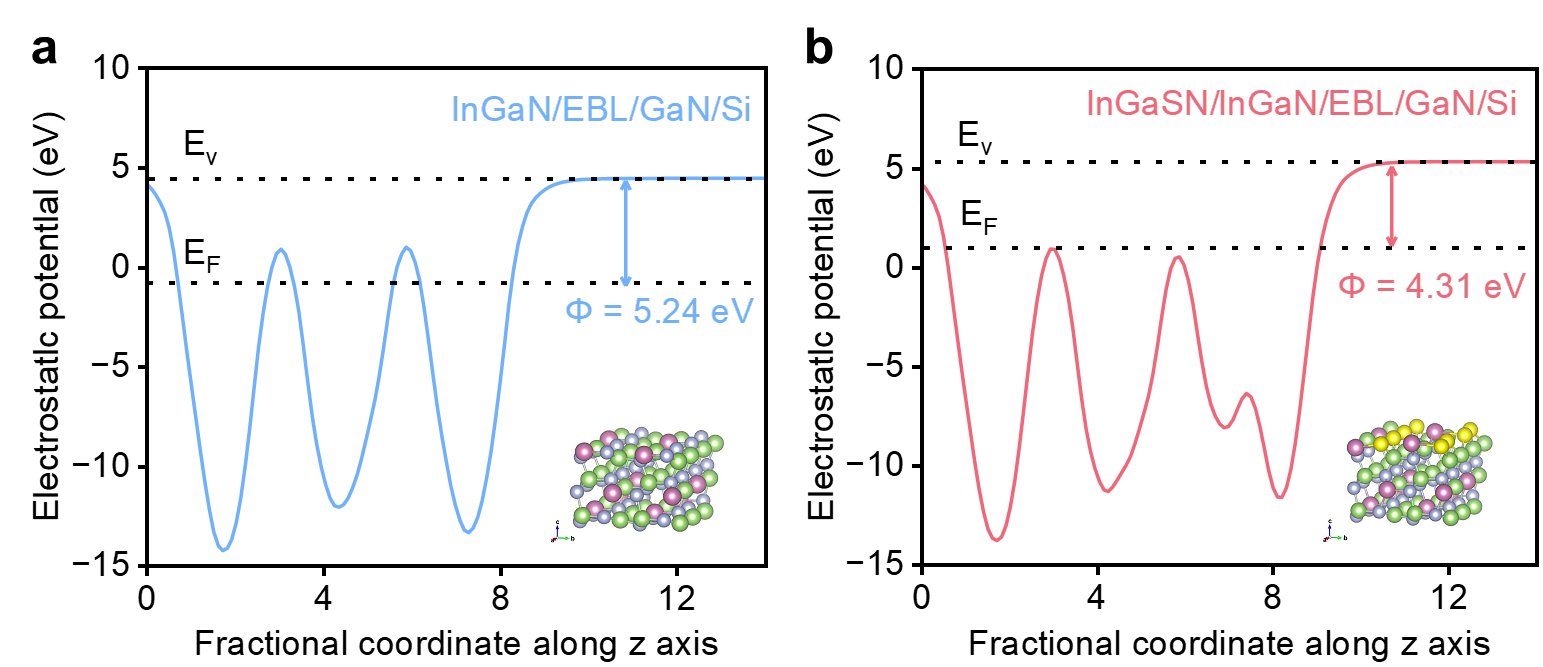
**

**Fig. S15** Calculated electrostatic potentials for **a** the InGaN/EBL/GaN/Si photocathode and **b** the InGaSN/InGaN/EBL/GaN/Si photocathode


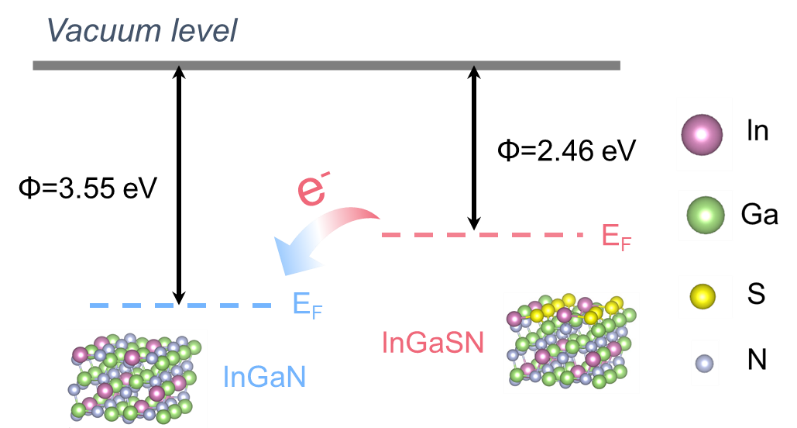


**Fig. S16** Schematic diagram of electron transfer between surface InGaSN and bulk InGaN


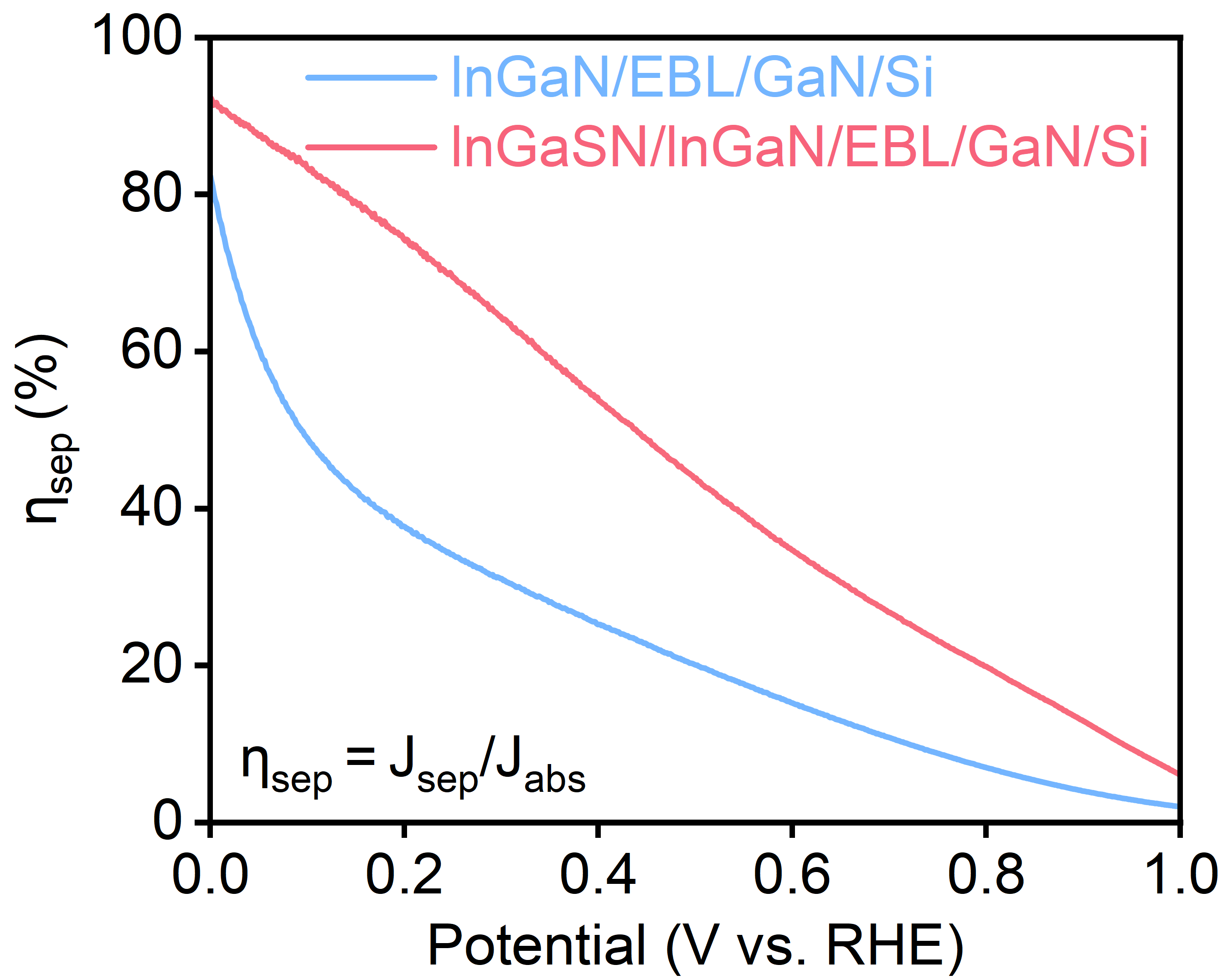


**Fig. S17** η_sep_ of InGaN/EBL/GaN/Si and /lnGaSNInGaN/EBL/GaN/Si


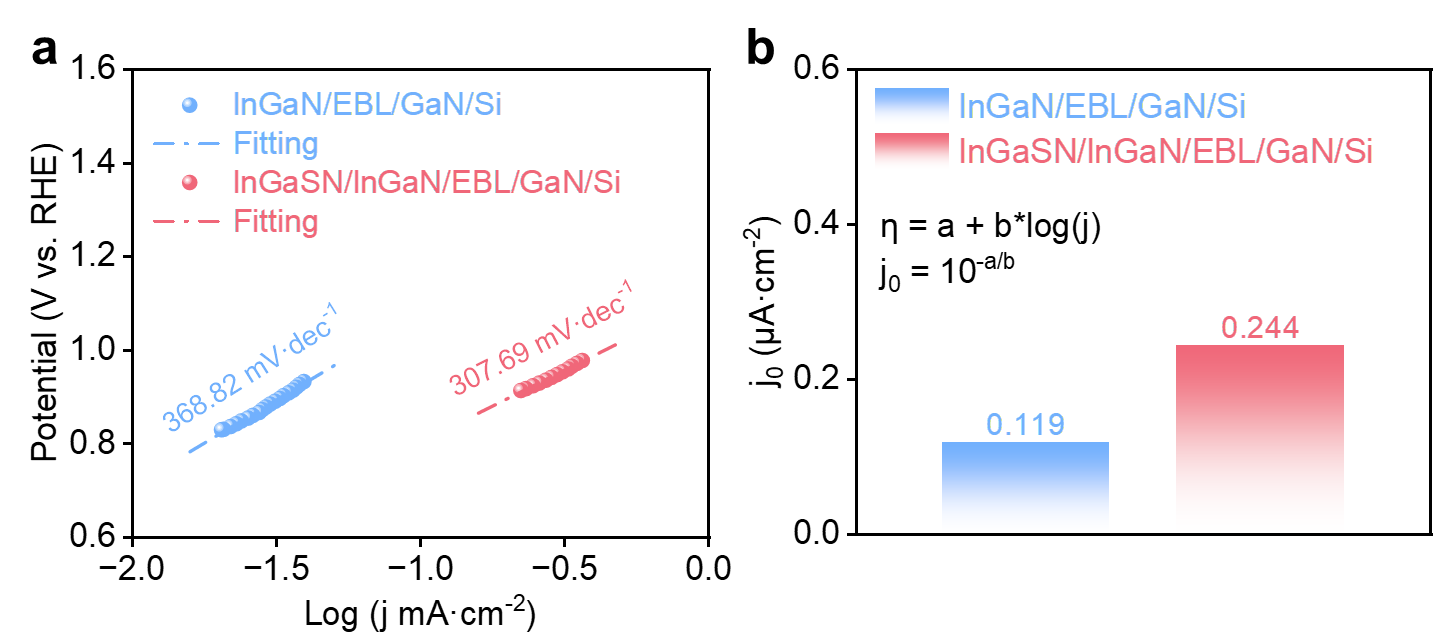


**Fig. S18** **a** Tafel slopes of lnGaN/EBL/GaN/Si and lnGaSN/lnGaN/EBL/GaN/Si. **b** Exchange current densities (j_0_) of lnGaN/EBL/GaN/Si and lnGaSN/lnGaN/EBL/GaN/Si

**Supplementary Tables**

Table S1. A comparison of the PEC performance of InGaN /GaN/Si, InGaN/EBL/GaN/Si, and lnGaSN/InGaN/EBL/GaN/Si.

(All photocurrents are measured at 0 V vs. RHE and expressed in units of mA cm^-2^.)


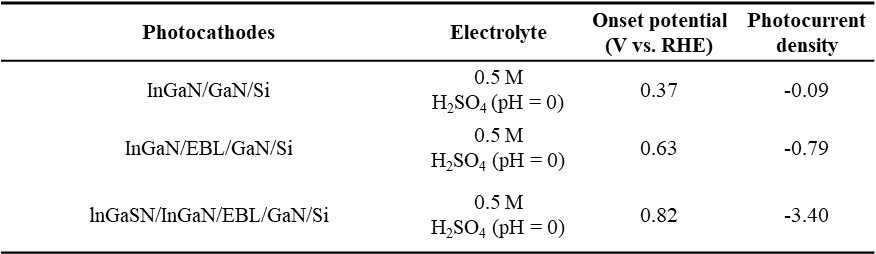


Table S2. A comparison of the PEC performance of this work to those of previously reported advanced semiconductor photocathode.

(All photocurrents are measured at 0 V vs. RHE and expressed in units of mA·cm^-2^.)


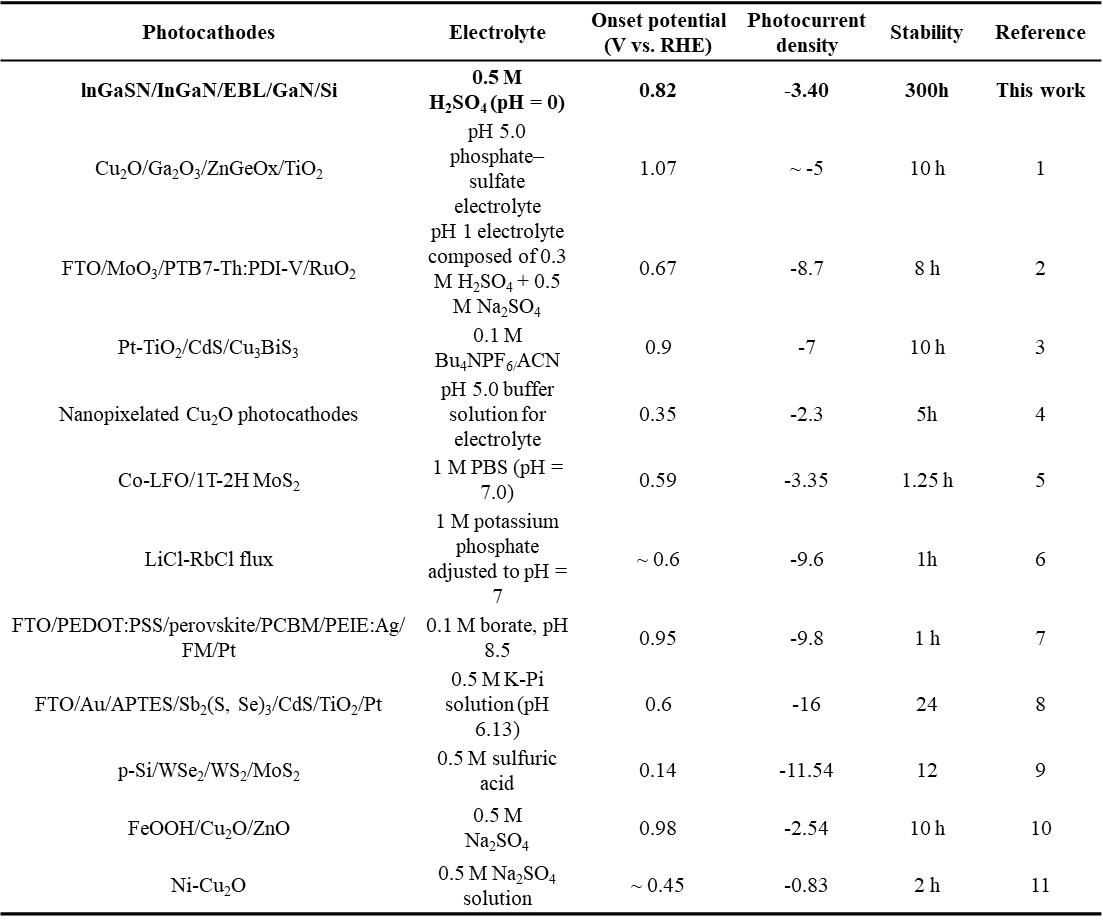


**Supplementary References**

1. J. Cheng, L. Wu, J. Luo, Improving the photovoltage of Cu_2_O photocathodes with dual buffer layers. Nat. Commun. **14**, 7228 (2023). <https://doi.org/10.1038/s41467-023-42799-x>
2. L. Yao, N. Guijarro, F. Boudoire, Y. Liu, A. Rahmanudin et al., Establishing Stability in Organic Semiconductor Photocathodes for Solar Hydrogen Production. J. Am. Chem. Soc. **142**, 7795-7802 (2020). <https://doi.org/10.1021/jacs.0c00126>
3. D. Huang, L. Li, K. Wang, Y. Li, K. Feng et al., Wittichenite semiconductor of Cu_3_BiS_3_ films for efficient hydrogen evolution from solar driven photoelectrochemical water splitting. Nat. Commun. **12**, 3795 (2021). <https://doi.org/10.1038/s41467-021-24060-5>
4. J. Lee, J. Oh, Nanopixelated Cuprous Oxide Photocathodes for Durable Photoelectrochemical Water Splitting. ACS Energy Lett. **7**, 3244-3250 (2022). <https://doi.org/10.1021/acsenergylett.2c01540>
5. X. Sun, Z. Hao, L. Cui, H. Li, L. Cao et al., 1T/2H MoS_2_ Functional Layer Boosts HER Kinetics of LaFeO_3_ Photocathode in Neutral Media through Hydrogen Spillover and Surface State Regulation. Adv. Funct. Mater. e12082 (2025). <https://doi.org/10.1002/adfm.202512082>
6. Y. Kageshima, T. Kanazawa, A. Uno, H. Kumagai, H. Minamisawa et al., Enhanced Photoelectrochemical Hydrogen Evolution over Crystalline Cu_2_Sn_0.38_Ge_0.62_S_3_ Particles Grown from LiCl-RbCl Flux. ACS Catal. 15, 4892-4900 (2025). <https://doi.org/10.1021/acscatal.5c00351>
7. M. C. Quesada, L. M. P. Outón, J. Warnan, M. F. Kuehnel, R. H. Friend et al., Metal-encapsulated organolead halide perovskite photocathode for solar-driven hydrogen evolution in water. Nat. Commun. **7**, 12555 (2016). <https://doi.org/10.1038/ncomms12555>
8. S. Lee, H. Lee, Y. S. Park, J. Yun, S. Moon et al., Self-assembled monolayer mediated fast charge transport of Sb_2_(S,Se)_3_ photocathode enabling high-performance unbiased water splitting. Nano Energy **126**, 109647 (2024). <https://doi.org/10.1016/j.nanoen.2024.109647>
9. S. Seo, S. Kim, H. Choi, J. Lee, H. Yoon et al., Direct In Situ Growth of Centimeter-Scale Multi-Heterojunction MoS_2_/WS_2_/WSe_2_ Thin-Film Catalyst for Photo-Electrochemical Hydrogen Evolution. Adv. Sci. **6**, 1900301 (2019). <https://doi.org/10.1002/advs.201900301>
10. S. Huai, X. Li, P. Li, S. Zhang, X. Huang et al., Rapid Charge Extraction via Hole and Electron Transfer Layers on Cu_2_O Photocathode for Stable and Efficient Photoelectrochemical Water Reduction. Adv. Sci. **12**, e09030 (2025). <https://doi.org/10.1002/advs.202509030>
11. M. Zhang, D. J. Wang, H. Xue, D. J. Zhang, P. D. S. Peng et al., Acceptor-Doping Accelerated Charge Separation in Cu_2_O Photocathode for Photoelectrochemical Water Splitting: Theoretical and Experimental Studies. Angew. Chem. **59**, 18463-18467 (2020). <https://doi.org/10.1002/anie.202007680>
